# Performance of community health workers under integrated community case management of childhood illnesses in eastern Uganda

**DOI:** 10.1186/1475-2875-11-282

**Published:** 2012-08-20

**Authors:** Joan N Kalyango, Elizeus Rutebemberwa, Tobias Alfven, Sarah Ssali, Stefan Peterson, Charles Karamagi

**Affiliations:** 1 Department of Public Health Sciences, Division of Global Health (IHCAR), Karolinska Institutet, SE 17177, Stockholm, Sweden; 2 Clinical Epidemiology Unit, Makerere University College of Health Sciences, P.O. Box 7072, Kampala, Uganda; 3 Department of Pharmacy, Makerere University College of Health Sciences, P.O. Box 7072, Kampala, Uganda; 4 Department of Health Policy, Planning and Management, School of Public Health, Makerere University College of Health Sciences, P.O. Box 7072, Kampala, Uganda; 5 Department of Paediatrics, Sach’s Children’s Hospital, Södersjukhuset, Stockholm, Sweden; 6 Department of Gender and Women Studies, Makerere University, P.O. Box 7072, Kampala, Uganda; 7 International Maternal and Child Health, Department of Women and Children’s Health, Uppsala University, Uppsala, Sweden; 8 Department of Paediatrics and Child Health, Makerere University College of Health Sciences, P.O. Box 7072, Kampala, Uganda

**Keywords:** CHW, ICCM, Health system research, Performance, Malaria, Pneumonia, Children, CMDs

## Abstract

**Background:**

Curative interventions delivered by community health workers (CHWs) were introduced to increase access to health services for children less than five years and have previously targeted single illnesses. However, CHWs in the integrated community case management of childhood illnesses strategy adopted in Uganda in 2010 will manage multiple illnesses. There is little documentation about the performance of CHWs in the management of multiple illnesses. This study compared the performance of CHWs managing malaria and pneumonia with performance of CHWs managing malaria alone in eastern Uganda and the factors influencing performance.

**Methods:**

A mixed methods study was conducted among 125 CHWs providing either dual malaria and pneumonia management or malaria management alone for children aged four to 59 months. Performance was assessed using knowledge tests, case scenarios of sick children, review of CHWs’ registers, and observation of CHWs in the dual management arm assessing respiratory symptoms. Four focus group discussions with CHWs were also conducted.

**Results:**

CHWs in the dual- and single-illness management arms had similar performance with respect to: overall knowledge of malaria (dual 72%, single 70%); eliciting malaria signs and symptoms (50% in both groups); prescribing anti-malarials based on case scenarios (82% dual, 80% single); and correct prescription of anti-malarials from record reviews (dual 99%, single 100%). In the dual-illness arm, scores for malaria and pneumonia differed on overall knowledge (72% *vs* 40%, p < 0.001); and correct doses of medicines from records (100% *vs* 96%, p < 0.001). According to records, 82% of the children with fast breathing had received an antibiotic. From observations 49% of CHWs counted respiratory rates within five breaths of the physician (gold standard) and 75% correctly classified the children. The factors perceived to influence CHWs’ performance were: community support and confidence, continued training, availability of drugs and other necessary supplies, and cooperation from formal health workers.

**Conclusion:**

CHWs providing dual-illness management handled malaria cases as well as CHWs providing single-illness management, and also performed reasonably well in the management of pneumonia. With appropriate training that emphasizes pneumonia assessment, adequate supervision, and provision of drugs and necessary supplies, CHWs can provide integrated treatment for malaria and pneumonia.

## Background

Efforts to reduce mortality among children less than five years, especially in resource-limited settings, have led to the introduction of community-based interventions to complement the formal health care systems for the treatment of common conditions in this age group [1]. Community-based interventions are meant to provide prompt and appropriate treatment for ill children in the community and are delivered by lay people commonly known as community health workers (CHWs). CHWs are persons selected from the communities where they live and work, and who undergo short-term training [1]. CHWs offer easy access to health services especially in rural or hard-to-reach areas. They have been used successfully in vertical programmes targeting single diseases, mainly for treatment of malaria [2-4] and in pilot studies for treatment of pneumonia [5,6].

Recognizing that many children suffer from or present with symptoms suggestive of multiple illnesses [7,8], it is recommended that CHWs should manage multiple childhood conditions through the integrated community case management of childhood illnesses (ICCM) [9]. Uganda adopted the ICCM policy, which in addition to promoting interventions in newborns, addresses the curative management of malaria, pneumonia, and diarrhea. These illnesses are the leading causes of death in children less than five years in Uganda, accounting for about 50% of deaths [10].

The management of multiple conditions by CHWs is likely to create challenges due to the increased complexity of the algorithm to be followed in the diagnosis and treatment of multiple illnesses, requiring wider knowledge and skill [11]. In addition to the assessment of fever, the CHWs have to assess respiratory symptoms including cough, respiratory rates, and difficulty in breathing. Assessment of respiratory symptoms in children is difficult [12]. Furthermore, the prescription of drugs in ICCM will be more complex because apart from the anti-malarials, they also prescribe antibiotics, and for treatment of diarrhea, oral rehydration salts and zinc.

A few studies have assessed CHW performance in the management of multiple conditions [11-14]. However, the results from these studies have been inconclusive and in some cases, the performance of CHWs has been suboptimal [11]. These studies, though few, seem to suggest that CHW performance in management of multiple illnesses among children is likely to be lower than CHW management of single illnesses. The aim of this study therefore was to compare the performance of CHWs in the dual management of malaria and pneumonia *vs* CHW management of malaria alone in children under five and to assess the factors influencing CHW performance.

## Methods

### Study design and setting

A mixed methods study with quantitative and qualitative data collection was conducted from June to July 2011 in Iganga-Mayuge Health and Demographic Surveillance Site (HDSS). The HDSS consists of 65 villages and is located in eastern Uganda, about 115 km from Kampala, the capital. The area is served by 131 CHWs known in the area as “community medicine distributors” (CMDs) who treat children aged less than five years. The CHWs complement the health services provided by the 10 government and three non-governmental health facilities and the 122 drug shops and private clinics. The CHWs have been providing health services in the area since 2009 under a cluster randomized trial (Trial registration number: ISRCTN52966230).

### Description of cluster randomized trial

The 65 villages of the HDSS are divided into intervention (dual-illness management) and control (single-illness management) areas and all villages have two CHWs each, except one village which has three CHWs because it is larger. The CHWs in the dual-management areas treat children aged 4–59 months with non-severe malaria and pneumonia using pre-packaged anti-malarials (artemether-lumefantrine, AL) and antibiotics (amoxicillin), respectively. Children with severe or other illnesses are referred to nearby health facilities. The CHWs in the single-management areas treat only children with non-severe malaria using anti-malarials. Children with respiratory or other symptoms or those with severe disease are referred to health facilities. The CHWs in the dual- and single-illness management areas do not treat children with diarrhea even though it is one of the illnesses targeted by the ICCM strategy because the implementation of the cluster randomized trial commenced in 2008 before the ICCM strategy was adopted in Uganda and was mainly informed by studies that had shown symptom overlap between malaria and pneumonia.

The CHWs diagnose malaria and pneumonia based on the integrated management of childhood illness (IMCI) classifications of illness [15]. Specifically, children are classified as having “malaria” if they have fever or history of fever within the previous 24 hours; and as having “pneumonia” if they have cough and difficult breathing or fast breathing (≥50 breaths per minute in children aged four to 12 months and ≥40 breaths per minute in children 12 to 59 months). A diagnosis of severe disease is made if the child has any of the four general danger signs: convulsions, repeated vomiting, lethargy/unconsciousness or failure to feed, or other danger signs: chest in-drawings, noisy breathing, severe dehydration and pallor. CHWs should follow up children that have been treated and refer those that did not get well to the nearest health unit. The CHWs do not use rapid diagnostic tests (RDTs) inspite of the 2010 WHO recommendation for malaria parasite based diagnosis [16] because the trial commenced before the recommendation was made. It is important to note that RDTs may not be available all the time in resource limited settings and CHWs may have to treat children without them.

The anti-malarials (artemether 20 mg, lumefantrine 120 mg) that CHWs use are available in two age-specific doses i.e., six tablets in a yellow pack for children aged less than 36 months and 12 tablets in a blue pack for children aged 36–59 months. The antibiotics (amoxicillin 125 mg) are available in three age-specific doses: six tablets (pink pack) for children less than 12 months, 12 tablets (green pack) for children aged 12-35 months, and 18 tablets (red pack) for children aged 36-59 months.

All CHWs received training on malaria for three days and those in the dual-management arm received a further three days training on acute respiratory illness (ARI). The training on malaria addressed signs and symptoms, danger signs, transmission, prevention, and populations at risk of malaria while that for ARI addressed signs and symptoms, use of respiratory timer, danger signs, and prevention of pneumonia. All CHWs were trained on referral, filling in registers, managing drug supplies, counseling caregivers of children, and adverse reaction monitoring. The training of CHWs was reinforced at monthly meetings with the supervisors of the project and health workers. The CHWs also received monthly supervision by health workers from the nearest health facility who checked the treatment practices of CHWs, drug storage and record keeping. The details of the cluster randomized trial have been described elsewhere [17].

### Participants

One hundred twenty five (125) of the 131 CHWs in Iganga-Mayuge HDSS that were available during the study period and who gave informed consent to participate were enrolled in the study.

### Data collection

A sequential explanatory approach which used qualitative findings to assist in explaining and interpreting the quantitative findings was used in the mixed methods data collection [18]. The quantitative data was collected first and was followed by the qualitative data after identifying areas that needed elaboration.

### Quantitative data

The quantitative data collection employed a multi-method approach that comprised of questionnaires, record reviews, follow up of children treated, and observation of CHWs in the dual-management arm.

The questionnaires were translated into the main local language of the area (Lusoga) and used to collect data on: socio-demographics, training received before commencement of CHW roles, continued training, support supervision, perceptions of community appreciation and support, and knowledge about malaria and pneumonia. In addition, five case scenarios presenting children of different age groups and symptoms were presented to evaluate the CHWs’ ability to elicit signs and symptoms, classify and respond to illness, prescribe medicines and give instructions (Additional file 1: Appendix 1). Unprompted rather than prompted questions were used due to their higher accuracy in measuring knowledge [19]. Knowledge of malaria and pneumonia had one question each that assessed the signs, transmission, prevention and danger signs [19] (Additional file 2: Appendix 2). The CHWs were probed and allowed to give more than one answer. A percentage score was computed to cater for having different total scores for each item [20,21]. CHWs in the single-illness management area were not assessed on pneumonia. Responses to the knowledge questions were marked against the information given to the CHWs during training as detailed in the training guide (Additional file 3: Appendix 3).

Record reviews of CHWs’ registers were used to evaluate their case-load over three months’ record keeping, and correctness of artemether-lumefantrine doses. In the dual-illness management arm the records were also checked for correctness of amoxicillin doses and correctness of prescription based on breathing rates recorded. The CHWs’ stock boxes were also checked. In addition, two children treated by the CHW in the week prior to the interview were randomly selected from the CHW’s register. Their caregivers were interviewed about the children’s presenting symptoms, demographics and the caregivers’ perceptions of the care provided.

CHWs in the dual-management arm were also observed assessing for respiratory symptoms of one child each at the nearest health facility. The observations were conducted by Medical Officers (holders of Bachelor of Medicine and Bachelor of Surgery degree) with training in IMCI. The CHWs counted the breathing rate (in parallel with the Medical Officer (gold standard)), assessed for chest in-drawing and classified the child as having “pneumonia” or not.

Data on CHWs’ socio-economic status (wealth index) and distance from the nearest health facility were extracted from the database of the HDSS.

### Outcome variable definition

The outcome measure was CHW performance defined as the ability to identify and respond to danger signs, elicit signs and symptoms, prescribe medicines (dosing, medicine administration instructions), and store medicines appropriately. These aspects were measured through knowledge assessment questions, case scenarios of children with different symptoms, and review of records of children treated by the CHWs [22]. Although high knowledge does not always transform into better performance, it is nevertheless an important prerequisite for one’s ability to perform a task [22]. Scores were generated using both principal components analysis (PCA) and mean percentages of the different items assessed [20,21]. The mean percentage scores gave each item assessed a weight of one [23]. The scores generated by both methods for the different items had fair to high correlations (0.64–0.88). The percentage scores are presented in this paper due to their ease of interpretability and comparability with other studies [23]. The Cronbach’s alpha for the items used in the score generation was overall 0.71.

### Qualitative data

The qualitative data was collected through focus group discussions (FGDs) moderated by an experienced qualitative researcher who was fluent in English and Lusoga (the main local language spoken in the area). The FGDs were conducted in Lusoga and were tape recorded. Four FGDs were held with CHWs; two for the dual- and two for the single-illness management area. The FGDs were conducted separately for males and females to allow free expression of the participants. Each FGD had eight to 10 participants who were selected by purposive sampling. The leaders of CHWs in both the dual- and single-illness management arms were selected together with other persons that were either active in the CHW meetings, had high patient turnover, had low patient turnover or had notable errors in their records or questionnaires based on preliminary evaluation of the quantitative data. The areas of focus for the FGDs were identified after preliminary analysis of the quantitative data and they included: training received before commencement of CHW roles and its adequacy, perceptions and support of the community towards the programme, referral practices, effects of current CHW roles on their lifestyle, and factors affecting the work of CHWs.

### Data management and analysis

The data were double entered in FoxPro computer package and analyzed using STATA 10 (STATA Corp, College Station, TX, USA). CHW characteristics and performance in the dual- and single- management areas were summarized using descriptive statistics and compared using chi-square or Fishers’ exact tests, and Mann Whitney U tests as appropriate. In addition, Wilcoxon signed rank tests were used to compare knowledge and performance on malaria and pneumonia management among the CHWs in the dual- management arm. The analysis of data from CHWs’ records was weighted by the number of children treated in the period of evaluation.

### Qualitative data

The qualitative data were transcribed and translated into English, and were analyzed using manifest content analysis with Open Code version 3.6 to develop codes and categories [24]. The transcripts were read several times and meaning units were identified and used to generate the codes, which were subsequently grouped into categories.

### Ethical issues

Permission to conduct the study was obtained from Makerere University School of Public Health Higher Degrees Research and Ethics Committee and Uganda National Council of Science and Technology. Written informed consent was obtained from the participants of the quantitative study while verbal consent was obtained from the FGD participants.

## Results

The data sources used to generate the results have been summarized in Table 1.

**Table 1:** Summary of data sources for various results presented

**Results presented**	**Data source**
Socio-demographic characteristics of the CHWs (Table 2)	CHW questionnaire
Training, supervision, and workload of CHWs (Table 3)	CHW questionnaire, CHW FGDs, CHWs’ records
Knowledge of malaria and pneumonia by CHWs (Table 4)	CHW questionnaire (knowledge tests)
Performance of CHWs in dual- and single-illness management arms based on case scenarios (Figure 1)	CHW questionnaire (case scenarios)
Performance of CHWs in dual- and single-illness management arms based on record reviews (Table 5)	Review of CHW records
Performance of CHWs in dual-illness management arm based on observation (Figure 2)	Observations of respiratory assessment in dual- management arm
Comparison of malaria and pneumonia management in dual-illness management arm* (Figure 3)	CHW questionnaire (knowledge tests, case scenarios), review of records
Report of CHW performance by caregivers of treated children	Caregiver questionnaire, triangulation with CHW FGDs
Factors perceived to influence CHW performance	CHW FGDs

**Figure 1:**
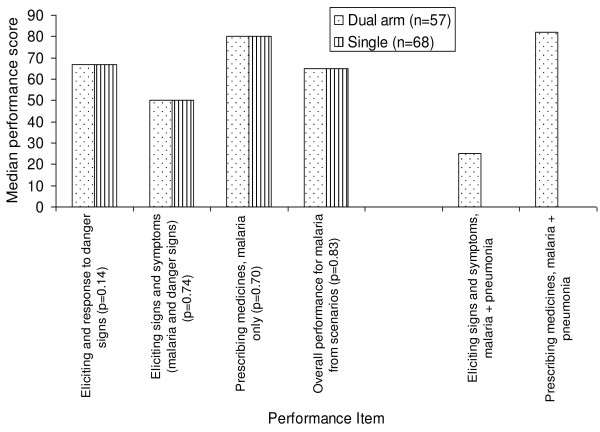
Performance of 125 Community Health Workers in Iganga-Mayuge HDSS based on case scenarios.

* Some of the results are also presented in Table 4, Figure 1, and Table 5 under the column for dual- illness management arm.

### Socio-demographic characteristics of CHWs

One hundred twenty five CHWs and 248 children were enrolled into the study in June 2011. The CHWs were mostly females, married, of the Anglican faith and had received secondary education at the level of senior one to four. The most common occupation was farming, and many CHWs had other programmes where they also worked as CHWs (62% dual-, 49% single-illness arm). Most CHWs were household heads and the majority had children less than five years living in their households. A higher percentage of CHWs in the dual- (11%) compared to the single- management arm (3%) had professional employment (p = 0.04). A majority of CHWs in both the dual- and single- management arms was in the highest two wealth quintiles (Table 2).

**Table 2:** Sociodemographic characteristics of 125 community health workers in Iganga-Mayuge HDSS

**Characteristic**	**Dual arm (n = 57)**	**Single (n = 68)**	**P-value**
Females, n (%)	34 (59.7)	40 (58.8)	0.93
Mean age (SD)	41.7 (8.3)	40.1 (9.5)	0.35
Religion, n (%)			
Catholic	5 (8.8)	2 (2.9)	0.18
Protestant	26 (45.6)	34 (50.0)	
Muslim	17 (29.8)	27 (39.7)	
Born again	9 (15.8)	5 (7.4)	
Education level, n (%)			
No formal education	1 (1.8)	0 (0)	0.16
Primary	13 (22.8)	12 (17.7)	
Secondary	38 (66.7)	55 (80.9)	
Tertiary	5 (8.8)	1 (1.5)	
Marital status, n (%)			
Married/Cohabiting	49 (86.0)	55 (80.9)	0.90
Single	3 (5.3)	5 (7.4)	
Divorced/Separated	2 (3.5)	3 (4.4)	
Widowed	3 (5.3)	5 (7.4)	
Occupation *, n (%)			
None	1 (1.8)	3 (4.4)	0.40
Farming	30 (52.6)	47 (69.1)	0.06
Trading	20 (35.1)	14 (20.6)	0.07
Housewife	5 (8.8)	3 (4.4)	0.32
Professional employment	7 (10.5)	2 (2.9)	0.04
Laborer/wage earner	2 (3.5)	0 (0)	0.12
Have other programme where currently CHW, n (%)	35 (61.5)	33 (48.5)	0.15
Median number of programmes where currently CHW (min, max)	2 (1,5)	1 (1,5)	0.19
CHW heads household, n (%)	32 (56.1)	40 (58.8)	0.76
CHW owns house where stays, n (%)	26 (45.6)	33 (48.5)	0.28
Mean number of people in household (SD)	7.3 (3.6)	7.1 (3.4)	0.86
Household has under-fives, n (%)	43 (75.4)	52 (76.5)	0.89
Mean number of under-fives (SD)	2.0 (1.0)	1.7 (0.9)	0.12
Median distance to nearest government health unit (min, max)	1.81 (0.12, 4.66)	1.49 (0.04, 4.71)	0.21
Median distance to nearest NGO health unit (min, max)	6.97 (0.73, 13.23)	6.57 (0.16, 14.67)	0.09
Wealth index, n (%)#			
Poorest	3 (6.4)	3 (6.4)	0.69
Poorer	5 (10.6)	10 (21.3)	
Poor	7 (14.9)	6 (12.8)	
Less poor	18 (38.3)	14 (29.8)	
Least poor	14 (29.8)	14 (29.8)	

*multiple responses possible # missing values.

### Training, supervision and workload of CHWs

All CHWs reported that they had been trained and most of them considered the training sufficient (88% dual-, 94% single- management arm). From the qualitative findings, the CHWs felt that familiarity with malaria and having manuals and charts to refer to helped to support the training.

*“In my own opinion, we would be able to perform our duties well because malaria isn’t something new in our lives. .. By the time we came here for training, we had some small experiences from our own lives. To me the three days were substantial to enable us to do our work in the villages”,* FGD among male CHWs in dual- management arm.

However, CHWs in the dual- management arm stated that assessment of respiratory symptoms and filling of records presented specific problems.

*“The three days weren’t enough for us because we trained to treat malaria and pneumonia at the same time. When we were trained to treat pneumonia, it was a bit difficult for us”,* FGD among female CHWs in dual- management arm.

Most CHWs had received support supervision from health workers. Some CHWs felt the workload was too heavy (23% dual, 28% single). The median number of children treated in the last three months was 80 in the dual- and 90 in the single- management arm. The quantitative results are summarized in Table 3.

**Table 3:** Training, supervision and workload of 125 community health workers in Iganga-Mayuge DSS

**Characteristic**	**Dual arm (n = 57)**	**Single (n = 68)**	**P-value**
Received training before started CHW work, n (%)	57 (100)	68 (100)	
Feel training received was sufficient, n (%)	50 (87.7)	64 (94.1)	0.21
Median months since last attended CHW meeting (min, max)	1 (0, 2)	0.75 (0,14)	0.10
Feel CHW meetings are useful, n (%)	57 (100)	67 (98.5)	0.36
Receive support supervision, n (%)	55 (96.5)	67 (98.5)	0.46
Median months since last support supervision (min, max)	1 (0,10)	1 (0,12)	0.92
Opinion about workload, n (%)			
Reasonable	41 (71.9)	44 (64.7)	0.75
Too little	3 (5.3)	5 (7.3)	
Too much	13 (22.8)	19 (27.9)	
Actions taken when away, n (%)			
Give to family member	0 (0)	1 (1.5)	0.63
Lock drugs up until return	54 (94.7)	64 (94.1)	
Ask fellow CHW to treat	3 (5.3)	3 (4.4)	
Median number of children treated in last 3 months (min, max)	80 (26, 126)	90 (30, 175)	0.07

From the qualitative findings, some CHWs had managed to organize their CHW roles around their other activities so that they were not affected by the workload.

*“Personally what I did, I wrote my mobile telephone number on the door so whenever the locals need me to treat the children when I am not around, they just get the number and they call me that they are at my home they have brought a sick child. We are not affected in any way”,* FGD among female CHWs in dual- management arm.

### Knowledge of malaria and pneumonia

More than 90% of CHWs mentioned fever as a sign of malaria; however the proportion was lower in the dual- (93%) compared to the single- management arm (100%). A high proportion of CHWs knew how malaria is transmitted (91% dual-, 94% single-). Most of the CHWs knew that insecticide-treated nets (ITNs) can prevent malaria (86% dual-, 91% single-), however, the overall knowledge of malaria prevention was moderate. Almost all CHWs could mention at least one danger sign (100% dual-, 99% single-). There was no difference in overall knowledge of malaria and danger signs between the dual- and single- management arms (72% *vs* 70% respectively, p = 0.37).

The median knowledge score of pneumonia signs among CHWs in the dual- management arm was 60% while that of pneumonia prevention was 20% (Table 4).

**Table 4:** Knowledge of malaria and pneumonia by community health workers in Iganga-Mayuge DSS

**Characteristic**	**Dual arm (n = 57)**	**Single (n = 68)**	**P-value**
Mention fever as sign of malaria, n (%)	53 (93.0)	68 (100)	0.03
Knowledge score on six malaria signs ¶			
Mean	54.1	52.5	0.64
SD	21.2	17.6	
Know malaria transmission, n (%)	52 (91.2)	64 (94.1)	0.53
Know ITNs as malaria prevention method, n (%)	49 (86.0)	62 (91.2)	0.36
Malaria prevention knowledge score ¶			
Median	57.1	42.9	0.46
min, max	14.3, 71.4	14.3, 71.4	
Overall malaria knowledge score			
Mean	64.5	63.4	0.67
SD	15.7	11.4	
Knowledge score of four general danger signs			
Median	75.0	75.0	0.47
min, max	0, 100	0, 100	
Mention at least one danger sign, n (%)	57 (100)	67 (98.5)	0.36
Malaria and danger signs knowledge score			
Median	71.9	69.8	0.37
Min, max	22.9, 93.2	18.2, 93.8	
**Pneumonia knowledge in dual management arm**			
Pneumonia signs knowledge score			
Median	60		
min, max	20, 100		
Pneumonia prevention knowledge score			
Median	20		
Min, max	0, 80		
Overall pneumonia knowledge score			
Median	40		
Min, max	10, 80		

### Performance of CHWs in dual- and single- management arms based on case scenarios

The median score on eliciting and responding to danger signs was 67% among CHWs in both dual- and single- management arms. The median score on eliciting signs and symptoms related to malaria was 50% in both arms. However, when signs and symptoms of pneumonia were included in the score for the dual- management arm, their median score reduced to 25%. The median score on prescribing medicines for malaria was 80% in both arms. There was no difference in the overall median score on malaria using case scenarios (65% in both groups). The median score on prescribing for both malaria and pneumonia in the dual- management arm was 82% (Figure 1).

### CHW performance based on review of their records

There was no difference in the proportion of CHWs with complete records, proportion supervised and the mean percentage of children that received correct doses of artemether-lumefantrine in the dual- (99%) and single- (100%) management arms. The mean percentage of children that received correct amoxicillin doses in the dual- management arm was 96% while the mean percentage of children with fast breathing that received amoxicillin was 82%. The mean percentage of children without fast breathing that received amoxicillin was 12% (Table 5). The errors with dosing of artemether-lumefantrine and amoxicillin were mostly among children whose ages were close to cut-offs at which dosing changed (usually within three months of the limit). In addition, there were errors in dosing of amoxicillin among children in the 12–35 months age-group where many of them were given the lower dose for less than 12 months.

**Table 5:** Results from review of 125 community health workers’ records in Iganga-Mayuge HDSS

**Characteristic**	**Dual arm (n = 57)**	**Single (n = 68)**	**P-value**
Record completeness, n (%)*	47 (82.5)	59 (86.8)	0.12
Supervised, n (%)	54 (94.7)	61 (89.7)	0.37
Artemether-lumefantrine correct dose (mean, SD)*	98.5 (4.9)	99.9 (0.7)	0.06
Amoxicillin correct dose (mean, SD)*	96.0 (7.5)		
With fast breathing given amoxicillin, mean (SD)*	81.7 (22.9)		
No fast breathing given amoxicillin, mean (SD)*	12.1 (22.2)		
Of those receiving amoxicillin, no fast breathing, mean (SD)*	9.3 (19.7)		
Storage box contains inappropriate materials, n (%)	5 (8.8)	0 (0)	0.01

* Results weighted by the number of children treated by the CHW.

### Performance of CHWs in dual- management arm based on observation of respiratory assessment

**Figure 2:**
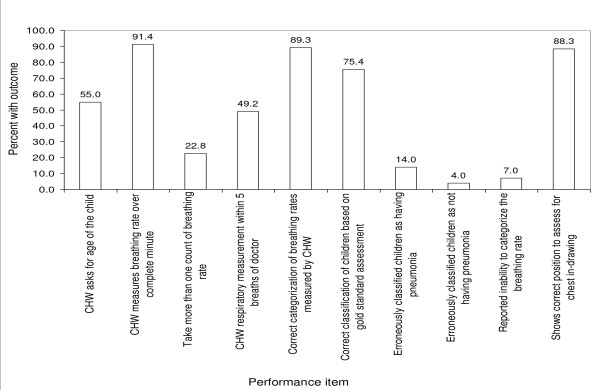
Respiratory assessment by Community Health Workers (n = 57) in the dual-illness management arm.

About 91% of CHWs measured the breathing rate over one minute and most of them (77%) took one measurement. Forty-nine percent and 39% had measurements within five and three breaths of those of the doctor, respectively. The majority of CHWs (89%) correctly categorized the breathing rates they had obtained into whether they indicated that a child had pneumonia or not even though only 55% of them asked for the age of the child. This may have been because only a small number of children had a breathing rate where the age would have played a role in the diagnosis. When the classification of children by the CHWs was compared to that of the gold standard, 75% of CHWs correctly classified the children as either having pneumonia or not, 14% misclassified children as having pneumonia, 4% misclassified children as not having pneumonia, while 7% said they could not decide if the child had pneumonia or not based on the breathing rate obtained. About 88% of CHWs were able to show the correct position for evaluation of chest in-drawing (Figure 2).

### Comparison of malaria and pneumonia knowledge and management in dual- management arm

**Figure 3:**
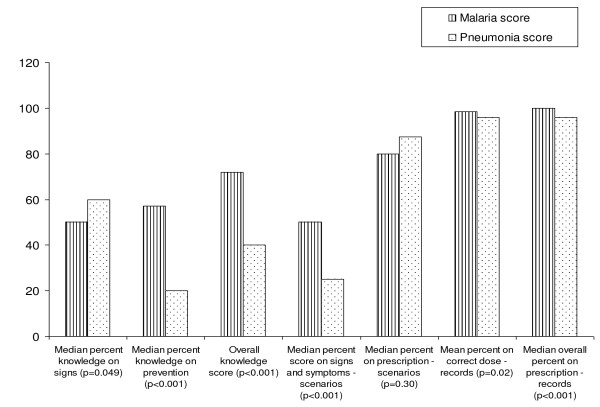
Comparison of malaria and pneumonia performance scores of CHWs in the dual-illness management arm from record reviews, case scenarios and knowledge questions (n = 57).

The CHWs in the dual- management arm had slightly higher knowledge on signs of pneumonia than signs of malaria (median 60% *vs* 50%, respectively). However, knowledge of malaria prevention (57%) was higher than knowledge of pneumonia prevention (20%) and the overall knowledge score for malaria was higher than that for pneumonia (median 72% *vs* 40% respectively, p < 0.001). The scores on prescription from case scenarios and records were high for both malaria and pneumonia (Figure 3).

### Report of CHW performance by caregivers of treated children

Data was collected from 248 caregivers instead of 250 because one CHW reported not knowing where all the children treated in the previous week lived. All the caregivers reported receiving instructions on how to administer the medicines. About 75% (86/113) in the dual- management arm and 73% (99/135) in the single- management arm reported being told what to do in case the child did not get well. The health care received from CHWs was rated as good (90% (103/113) dual-, 84% (113/135) single-) or fair (10% (11/113) dual-, 16% (21/135) single-) and only one person in the single- management arm (0.7%) rated the care as poor (p = 0.24). All caregivers in both arms were willing to seek care from the CHW again. The most common suggestions made by caregivers for improvement of CHW work were to: increase the number of drug types accessible through the CHWs (10% dual-, 33% single-), improve the stock management to avoid frequent stock outs (14% dual-, 17% single-), widen age range treated (2.7% dual-, 1.5% single-), give treatment in time (1.8% dual-, 1% single-), avail more equipment, e g, thermometers (2.7% dual-), and not delegating work to other persons in home (1.8% dual-). The suggestion to increase the age range of children treated was also reflected in the FGDs.

*“Sometimes the people say that it seems these health workers want to kill our children with those drugs reason being that why are we very strict on who we treat? They said that it would be good if everyone is treated. If possible we could increase the age group to 7 years”,* FGD among female CHWs in single- management arm.

### Factors perceived by CHWs to influence performance

From the qualitative findings, the factors perceived to influence CHW performance were grouped into community factors, CHW programme factors and health facility-related factors. The community factors included: mobilization of communities by the local leaders and confidence of the community in medicines used which enhanced performance; and caregivers’ non-compliance with referral and lack of community appreciation for age restrictions of children treated which impacted performance negatively.

*“The LCs* (local councils) (local leaders) *also play very important roles here. They tell people to come for treatment from me and as a result they come whenever the children are sick”,* FGD among female CHWs in single- management arm*.*

The CHW programme factors that enhanced performance included: re-enforcement of knowledge through monthly meetings, availability of medicines, using safe medicines, and having transport refund that enabled them to collect the medicines (some CHWs however felt the transport refund was not sufficient). However, there were CHW programme factors that lowered performance including: lack of materials to enable them to perform their work at night and during the rainy weather, lack of transport for follow up of treated children, and having large coverage areas which complicated follow up of children.

*“When we don’t have drugs, everything goes down. Transport to do follow up of the children that you treated is really hard. Sometimes you even use your own personal money to do follow up”,* FGD among female CHWs in single- management arm.

The health facility-related factor influencing performance was the lack of cooperation from health workers at facilities.

*“There are times when you refer a person to the health centre but when this patient reaches there, he/she doesn’t get the needed attention. They say that they look at the referral note over and over again instead of treating the patient”,* FGD among female CHWs in single- management arm*.*

## Discussion

In this study, knowledge and performance of CHWs on malaria did not differ significantly between the dual- and single-illness management arms. Both arms had fairly high scores on knowledge of malaria (72% dual-, 70% single-); and high scores on prescribing by case scenarios (80% in both arms) and record reviews (99% dual-, 100% single-). The care received from CHWs was rated highly by caregivers in both arms (90% dual-, 84% single-). The factors perceived to influence CHW performance included community, CHW programme and health facility-related factors.

The similarity of the knowledge on malaria in the dual- and single-illness management arms suggests that the requirement to have knowledge of pneumonia may not impact negatively on knowledge of malaria. This could be due to long familiarity with malaria for this community, an argument supported by the FGD findings where the CHWs felt that training for malaria was adequate because malaria was not new in their community. The management of malaria at the community level has been in effect in Uganda since 2002 under the home-based management of fever strategy [25].

Although most CHWs mentioned fever as a sign of malaria (93% dual-, 100% single-), knowledge of other signs was quite low. Most CHWs mentioned ITNs among the malaria prevention methods but other methods were not well known. Knowledge of malaria transmission and danger signs was high. The results are comparable to a study among village malaria workers in Cambodia where knowledge of malaria signs was low [26]. However, in contrast to that study where only 19.5% knew malaria transmission, more than 90% of CHWs in the current study knew how malaria is transmitted. This considerable knowledge of CHWs on malaria prevention and transmission can be utilized in strengthening malaria prevention dissemination.

The performance of CHWs in the management of malaria from case scenarios was in agreement with that from knowledge assessment, showing no difference between the dual- and single-illness management arms (median 65% for both arms). This similarly implies that pneumonia management may not impact malaria management negatively. The performance on eliciting signs and symptoms for malaria based on case scenarios was generally low (50% for both arms) and that for combined malaria and pneumonia in the dual-illness arm was lower (25%). The findings in the current study are similar to those reported in a study in Kenya where the performance of CHWs in eliciting signs for malaria over three evaluations was 41-64% [11]. The findings of lower performance in eliciting signs and symptoms among children with pneumonia are similar to what was found in Kenya where the sensitivity of CHW classification of pneumonia was 31.5 to 54.5% [11].

As reported previously, the assessment of respiratory symptoms was difficult for the CHWs. This is supported by the reports from the FGDs that difficulties were experienced during the training for respiratory assessments and the quantitative findings where a higher proportion (although non-significant) of CHWs in the dual- management arm stated that the training received was not adequate. In addition, CHWs have been used to manage fever as a sign of malaria in Uganda since 2002 [25] and they are, therefore, more familiar with its signs and symptoms. In contrast, awareness of pneumonia is fairly new among the general population and its diagnosis involves a more complicated algorithm of counting breathing rates, assessment for noisy breathing and chest in-drawings. These tasks have been shown to create challenges [11,12]. The fewer number of cases of pneumonia treated by CHWs compared to malaria cases also provides fewer opportunities to improve skills in the treatment of pneumonia.

The high scores on correct prescriptions for malaria and pneumonia from case scenarios and record reviews for both the dual- and single-illness management areas are comparable to studies in Rwanda and Kenya. In Rwanda, the range of correct prescriptions for malaria was 78-99% and that for pneumonia from three districts was 85-100% [27] while in Kenya the correct treatment for malaria was 91% [11]. In the review of records, the correct dosing of amoxicillin, though very high (96%), was significantly lower than that of anti-malarials (99%, p = 0.009) in the dual- management arm. This is probably because the amoxicillin tablets used for pneumonia treatment have not been previously used in this setting while artemether-lumefantrine (anti-malarial) has been in use since 2005 [28]. Both artemether-lumefantrine and amoxicillin had errors in dosing mainly close to the thresholds for changes in the dose highlighting the need to emphasize the cut-off ages for the different doses during training and re-training. In addition, some children aged 12–35 months who should have received the twelve-tablet pack of amoxicillin received the six-tablet pack. This could have been due to occasional confusion of amoxicillin dosing with artemether-lumefantrine dosing since the latter has two pre-packed doses (<36 months and 36–59 months) while the former has three (<12 months, 12–35 months, and 36–59 months).

A high proportion of children recorded with fast breathing (82%) received antibiotics appropriately. This proportion is higher than what was found in another study in Uganda where only 40% of the children that needed antibiotics received them. However, similar to that study where 10% of the children without malaria or pneumonia received either antibiotics or anti-malarials, 12% of children without fast breathing in the current study received amoxicillin inappropriately [12]. The challenges in correct assignment of treatment to children are most likely due to difficulties in both counting and categorizing breathing rates. From observation of CHWs’ assessment of respiratory symptoms, 49% of the CHWs estimated breathing rates within five breaths of those of the doctors (gold standard) and an even smaller proportion (39%) estimated breathing rates within three units of those of the doctor. However, a higher proportion of CHWs (89%) were able to correctly categorize the breathing rates they obtained showing that the main problem may be in measurement of breathing rates. As a result, some CHWs may record inaccurate breathing rates for the sake of filling the register but instead use other criteria to treat the children. The challenges in respiratory assessment may result in misclassification of children’s illness. From observation of respiratory assessment, 14% of CHWs misclassified children as having pneumonia while 4% misclassified children as not having pneumonia compared to the gold standard assessment. This implies that although a child presenting with pneumonia symptoms will be more likely to get appropriate treatment, some children may miss treatment or be treated inappropriately. There are higher chances of children without pneumonia being treated for pneumonia than children with pneumonia not being treated for pneumonia. Children may be inappropriately treated with antibiotics due to pressure to treat with a particular drug. Nevertheless, a considerable number of children that would not have received prompt treatment for pneumonia symptoms if treatment were not integrated receive it. The proportion of CHWs with correct breathing counts in previous studies ranges from 42 to 80% [6,12,27]. The findings suggest a need for better procedures and tools to assess breathing rate and provide drugs like paracetamol to the CHWs which may help them deal with the pressure to treat children that may be febrile but who do not qualify for antibiotic treatment. Additional research is also needed to determine the extent to which integrated management of childhood illness improves coverage of correct management for pneumonia.

The factors perceived to influence performance by CHW are similar to what has been reported elsewhere through mainly quantitative but also qualitative studies. Training of health workers influences their performance [29]. Irregular supply of drugs was found to contribute to low performance in Zambia [30]. Community and financial support have also been cited as influencing performance [31]. Community support in the form of feedback and rewards was found to have greater influence on CHW performance than that from the health system [32]. Large population coverage has been found to lower performance of CHWs [33]. Many of the factors perceived by CHWs to influence performance in the current study can be addressed through increased sensitization of the community and health workers, and improvements in the CHW programmes especially regarding the drugs and supplies management. There was community sensitization about the programme before its implementation but not afterwards thereby missing the opportunity to re-enforce messages to the community. In addition, there were monthly meetings between health workers at the health facilities in Iganga-Mayuge HDSS and the project managers of the cluster randomized trial which provided opportunity for continued sensitization of the health workers. However since the CHWs would not attend the meetings, their concerns may not have been relayed to the health workers.

The drug supplies and management could be improved at the programme level through better quantification of drug needs and timely ordering of drugs taking into account the long and varied lengths of time needed to obtain drugs from different suppliers. The long lead times experienced with some suppliers would sometimes result in drug stock outs. In addition, CHWs should be trained on identifying minimum stock levels so that they can have timely ordering of drugs. Furthermore, flexible drug replenishment systems that take into account the variations in CHWs’ patient load should be devised.

### Methodological issues

This study is limited in using knowledge tests, case scenarios and record reviews of the CHWs’ registers to assess performance because these may not reflect their actual practice. However, these methods were able to standardize the cases, present various scenarios that may not have been encountered during the study period and assess competence in applying knowledge [22]. These methods do not assess skills well. However, the CHWs’ skills as they assessed children for respiratory symptoms were observed. In addition, the scenarios used may not have been adequate to comprehensively assess performance in assessing signs and symptoms because the length of the questionnaire had to be limited. The combination of several methods however, offers strength to this study. About 5% of the CHWs could not be contacted. These may have had different performance from those studied, but represent only a small part of the study population.

## Conclusion

CHWs in the dual- and single-illness management arms have similar knowledge and perform equally well in handling malaria cases. This implies that the requirement to have knowledge and take care of children with pneumonia does not impact negatively on knowledge and performance of handling malaria. Although the performance of CHWs in the management of malaria is higher than for pneumonia, the CHWs perform reasonably well in the management of pneumonia. The CHWs find challenges in identifying signs and symptoms of pneumonia, counting respiratory rates and categorizing them, and assigning children to the correct treatment. The main challenge though arises from assessment and classification of symptoms as opposed to prescription of medicines. The findings from this study suggest that CHWs can adequately provide integrated malaria and pneumonia management with appropriate support. The support should include adequate supervision and continued training that emphasizes the more difficult aspect of pneumonia signs, assessment, and treatment; and providing the necessary equipment and supplies, e.g., respiratory timers, drugs.

## Abbreviations

AL: Artemether-Lumefantrine; ARI: Acute respiratory illness; CHWs: Community Health Workers; CMDs: Community Medicines Distributors; FGD: Focus Group Discussion; HDSS: Health and Demographic Surveillance Site; ICCM: Integrated Community Case Management; IMCI: Integrated Management of Childhood Illnesses; ITNs: Insecticide Treated Nets; LC: Local Council; PCA: Principal Components Analysis.

## Competing interests

The authors declare that they have no competing interests.

## Authors’ contributions

JNK, ER, SP, CK, TA and SS took part in designing the study, development of study tools, data analysis and manuscript writing. JNK participated in data collection. All authors read and approved the final manuscript.

## Supplementary Material

Additional file 1 **Appendix 1.** Case scenarios of sick children. Click here for file

Additional file 2 **Appendix 2.** Knowledge questions for malaria and pneumonia signs, prevention and danger signs. Click here for file

Additional file 3 **Appendix 3.** Community Health Worker Training guide. Click here for file
