# Supplementary material for: Performance of community health workers under integrated community case management of childhood illnesses in eastern Uganda
Source: Malar J. 2012 Aug 20;11:282. doi: 10.1186/1475-2875-11-282 (PMC3480882; doi:10.1186/1475-2875-11-282)
Supplement: Additional file 1 — Appendix 1. Case scenarios of sick children. [file 1475-2875-11-282-S1.doc]

Appendix 1. Case scenarios of sick children

**Case scenario 1 presented a child with malaria and pneumonia. The purpose of this was to assess CHWs ability to elicit signs and symptoms, classify disease, and take appropriate action either by referring cases where appropriate (single management arm) or choose appropriate treatment (dual management arm).**

1) Jane’s mother brings her to see you with a fever. What is the first action that you would take? ***(DO NOT READ OPTIONS – Circle one)***

1. Treat Jane with Coartem

2. Treat Jane with Amoxycillin

3. Ask for Jane’s full name, age and whether this is her first visit for this episode of illness

4. Refer her to health facility

5. Other (specify) __________________________________________

2) You found out that Jane Kantono is 7 months old, she has been ill for one day with a fever and this is her first visit for this illness episode. What would you do next? ***(Circle all that apply – DO NOT READ OPTIONS)***

1. Ask if Jane has been vomiting repeatedly

2. Ask if Jane has had convulsions

3. Ask if Jane has been sleepy or drowsy

4. Ask if Jane has been unable to feed

5. Treat Jane with Coartem

6. Treat Jane with Amoxycillin

7. refer her to the health facility

8. Other (specify) __________________________________________________

3) You check Jane for general danger signs and there are none. You then ask the mother whether Jane has had cough and difficult breathing and she says ‘yes’. What would you do next? ***(Circle all that apply – DO NOT READ OPTIONS)***

1. Treat Jane with Coartem

2. Treat Jane with Amoxycillin

3. Refer her to the health facility

4. Ask the mother to raise the shirt to look for chest in-drawing

5. Listen for noisy breathing

6. Other (specify) __________________________________

4) Supposing you checked Jane for chest in-drawing and noisy breathing and you found that they were both not present, what would you do next? ***(Circle all that apply – DO NOT READ OPTIONS)***

1. Treat Jane with Coartem

2. Treat Jane with Amoxycillin

3. Refer her to the health facility

4. Count the respiratory rate (bala emirundi gyaisa omuuka buli dakiika)

5. Other (specify) __________________________________

5). If you decided to count Jane’s respiratory rate and found it at 55 breaths per minute on the first count and 57 breaths per minute on the second count. So now you have information that Jane Kantono, a 7 months old girl has been ill for one day with fever and this is her first visit for this episode of illness. She has no convulsions, no vomiting, is able to feed and is conscious. She has cough and difficult breathing but no chest in-drawing or noisy breathing. How would you classify Jane’s illness? **(*DO NOT READ OUT OPTIONS)***

1. Classify the illness as non-severe malaria alone

2. Classify the illness as severe malaria alone

3. Classify the illness as non-severe pneumonia alone

4. Classify the illness as severe pneumonia alone

5. Classify the illness as non-severe malaria and pneumonia

6. Classify the illness as severe malaria and pneumonia

7. Classify the illness as neither malaria nor pneumonia

8. Other (specify) _______________________________________

6) What actions would you take following your classification of Jane’s illness in question 58 above? ***(Circle all that apply – DO NOT READ OPTIONS)***

1. Treat Jane with Coartem alone

2. Treat Jane with Amoxycillin alone

3. Treat Jane with both Coartem and Amoxycillin

4. Refer Jane to a health facility

5. Ask Jane’s mother to take her home and only come back if the illness becomes more serious

6. Other (specify) _________________________________________

**Case scenario 2 presented a child with general danger signs. The purpose was to assess ability to identify and respond appropriately to general danger signs.**

1) Mukyala Judith a 2 year old child is brought by her mother to you. Judith has had a fever for one day. As you listen to the mother tell you about her illness, she vomits about 4 times. The mother also reports that Judith has been vomiting frequently since last night and has been very sleepy for the last 6 hours. What actions would you take? ***(Circle all that CHW mentions – DO NOT READ OPTIONS)***

1. Treat the child with Coartem (yellow pack)
2. Treat the child with Coartem (blue pack)
3. Refer the child to the nearest health facility
4. Treat the child with Amoxycillin
5. Ask the mother for any other symptoms before I decide on the action to take
6. Other (specify) ______________________________________

2) What are your reasons for the action(s) you have taken with Judith above? ***(Circle all that CHW mentions – DO NOT READ OPTIONS)***

1. This child clearly has malaria which I should treat with antimalarials

2. Because the child is vomiting repeatedly which is a danger sign

3. Because the child has been very sleepy which is a danger sign

4. This child clearly has both malaria and pneumonia which I should treat

5. I need to find out more symptoms to make a diagnosis

6. Other (specify) ______________________________________________

**Case scenario 3 presented a child with non-severe malaria and its purpose was to assess prescription of correct drugs and doses and give appropriate instructions to the caregiver.**

1) What treatment would you give Nabwire Rose a 9 month old child that you have classified as having non-severe malaria? ***(Circle all that CHW mentions – DO NOT READ OPTIONS)***

1. Coartem (yellow pack)
2. Coartem (blue pack)
3. Amoxycillin

4. Other (specify) _____________________

2) For the medicine chosen in question 62 above, what dose and frequency of medicine would you give Nabwire Rose? ***(Circle all that CHW mentions – DO NOT READ OPTIONS)***

1. Coartem (1 tablet once a day) 7. Amoxycillin (1 tablet once a day)

2. Coartem (1 tablet twice a day) 8. Amoxycillin (1 tablet twice a day)

3. Coartem (1tablet three times a day) 9 Amoxycillin (1 tablet three times a day)

4. Coartem (2 tablets once a day) 10 Amoxycillin (2 tablets once a day)

5. Coartem (2 tablets twice a day) 11 Amoxycillin (2 tablets twice a day)

6. Coartem (2 tablets three times a day) 12 Amoxycillin (2 tablets three times a day)

3) State the instructions you would give to Nabwire Rose’s caretaker ***(Circle all that CHW mentions – DO NOT READ OPTIONS)***

1. How to take the medicines

2. To take the medicines with a fatty meal

3. To complete the treatment

4. To take the child to a health facility if she did not get better

5. To come back for more treatment if the child did not get better

6. To continue breast feeding the child

7. Other (specify) _________________________________________________

**Case scenario 4 presented a child with malaria and pneumonia and its purpose was to assess prescription practices as well as correct response to children with pneumonia in the single management arm.**

1) Moustapha Wambuzi, a 48 months old boy is brought to you. He has a fever and he seems to be breathing fast. After examining him and asking for further information, you decide that Moustapha has non-severe malaria and pneumonia. What actions would you take? **(*Circle all that CHW mentions – DO NOT READ OPTIONS)***

1. Refer the child immediately to a health facility

2. Treat the child with Coartem (1 tablet once daily)

3. Treat with Coartem (1 tablet twice daily)

4. Treat the child with Coartem (2 tablets once daily)

5. Treat with Coartem (2 tablets twice daily)

6. Treat the child with Amoxycillin (1 tablet once daily)

7. Treat the child with Amoxycillin (1 tablet twice daily)

8. Treat the child with Amoxycillin (2 tablets once daily)

9. Treat the child with Amoxycillin (2 tablets twice daily)

10. Treat the child with Amoxycillin (3 tablet once daily)

11. Treat the child with Amoxycillin (3 tablet twice daily)

12. Other (specify) ___________________________________________________________

**Case scenario 5 presented a two months old child with fever. Its purpose was to assess CHWs ability to identify age groups of children that they treat.**

1) What treatment would you give Maria a 2months old child that has fever with no other symptoms? **(*Circle all that CHW mentions – DO NOT READ OPTIONS)***

1. Treat with Coartem (yellow pack)

2. Treat with Coartem (blue pack)

3. Treat with Coartem (yellow pack) and Amoxycillin (1 tablet twice a day)

4. Treat with Coartem (blue pack) and Amoxycillin (1 tablet twice a day)

5. Refer the child to health facility

6. Other (specify) _____________________________________
