# Supplementary material for: Performance of community health workers under integrated community case management of childhood illnesses in eastern Uganda
Source: Malar J. 2012 Aug 20;11:282. doi: 10.1186/1475-2875-11-282 (PMC3480882; doi:10.1186/1475-2875-11-282)
Supplement: Additional file 2 — Appendix 2. Knowledge questions for malaria and pneumonia signs, prevention and danger signs. [file 1475-2875-11-282-S2.doc]

Appendix 2. Knowledge questions for malaria and pneumonia signs, prevention and danger signs

1.What are the signs of malaria? ***(Circle all that respondent mentions – DO NOT READ OUT OPTIONS)***

1. Fever / hot body 5. Pallor 9. Vomiting

2. Chills 6. Body ache 10. Running nose

3. Sweating 7. Diarrhea 11. I don’t know

4. Headache 8. Loss of appetite 12. Other (specify) _____________

2.How is malaria passed from person to person? ***(Circle all that respondent mentions – DO NOT READ OUT OPTIONS)***

1. Sneezing 6. Sharing food 11. Other (specify) _________

2. Touch blood 7. Breathing same air 12. Other (specify) _________

3. Touch utensils 8. Getting close to someone with malaria

4. Mosquito bites 9. Sharing beddings

5. Coming close to mosquitoes 10. I don’t know

3.What information can you tell a caretaker to prevent malaria in children? ***(Circle all that respondent mentions – DO NOT READ OUT OPTIONS)***

1. Clear bushes around the house 6. Cover water tanks

2. Sleep under mosquito nets 7. Not sharing food

3. Spray house 8. Stay away from person that has malaria

4. Use mosquito coils 9. I don’t know

5. Fill in water puddles 10. Other (specify) ____________________

11. Other (specify) ____________________

4. What are the signs of a child with pneumonia? ***(Circle all that respondent mentions – DO NOT READ OUT OPTIONS)***

1. Fever 5. Chest in drawing 9. Diarrhea

2. Cough 6. Chills 10. I don’t know

3. Fast breathing 7. Loss of appetite 11. Other (specify) _____________

4. Noisy breathing 8. Pallor 12. Other (specify) _____________

5.What advice can you give a caretaker to prevent pneumonia in children? ***(Circle all that respondent mentions – DO NOT READ OUT OPTIONS)***

1. Adequate nutrition 7. I don’t know

2. Reduce indoor pollution 8. Other (specify) _____________________

3. Immunize children 9. Other (specify) _____________________

4. Breast feeding

5. Children should sleep under mosquito nets

6. Keep children away from excessive cold

6.Name signs of a severely sick child that you need to refer to the health facility ***(Circle all that respondent mentions – DO NOT READ OUT OPTIONS)***

1. Not able to drink or breast feed

2. Vomits everything

3. Has convulsions

4. Drowsiness or unconscious

5. Chest in drawing

6. Lack of blood (shown by pale lips or palms i.e. pallor)

7. Severe dehydration

8. Extreme weakness

10. Difficult or noisy breathing

11. Other (specify) _______________________

12. Other (specify) _______________________
