# Supplementary material for: Performance of community health workers under integrated community case management of childhood illnesses in eastern Uganda
Source: Malar J. 2012 Aug 20;11:282. doi: 10.1186/1475-2875-11-282 (PMC3480882; doi:10.1186/1475-2875-11-282)
Supplement: Additional file 3 — Appendix 3. Community Health Worker Training guide. [file 1475-2875-11-282-S3.doc]

**Appendix 3. Community Health Worker Training guide**

**
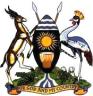
**

**COMMUNITY MEDICINE DISTRIBUTOR TRAINING GUIDE**

**ON THE**

**INTEGRATED MANAGEMENT OF MALARIA AND PNEUMONIA**


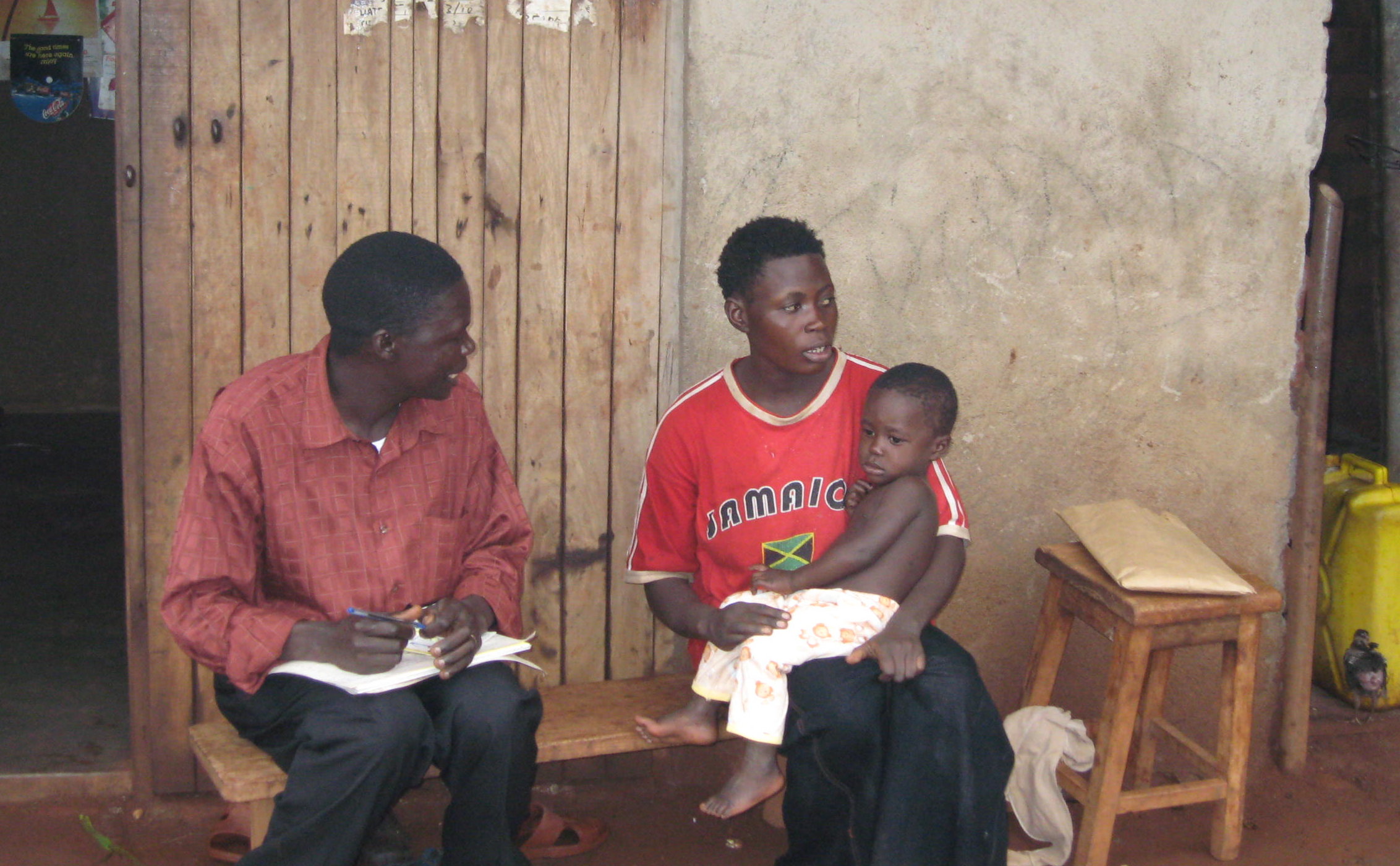


**INTERVENTION ARM**

**ADAPTED FROM THE ZAMBIA REFERENCE MANUAL FOR COMMUNITY HEALTH WORKERS**

#
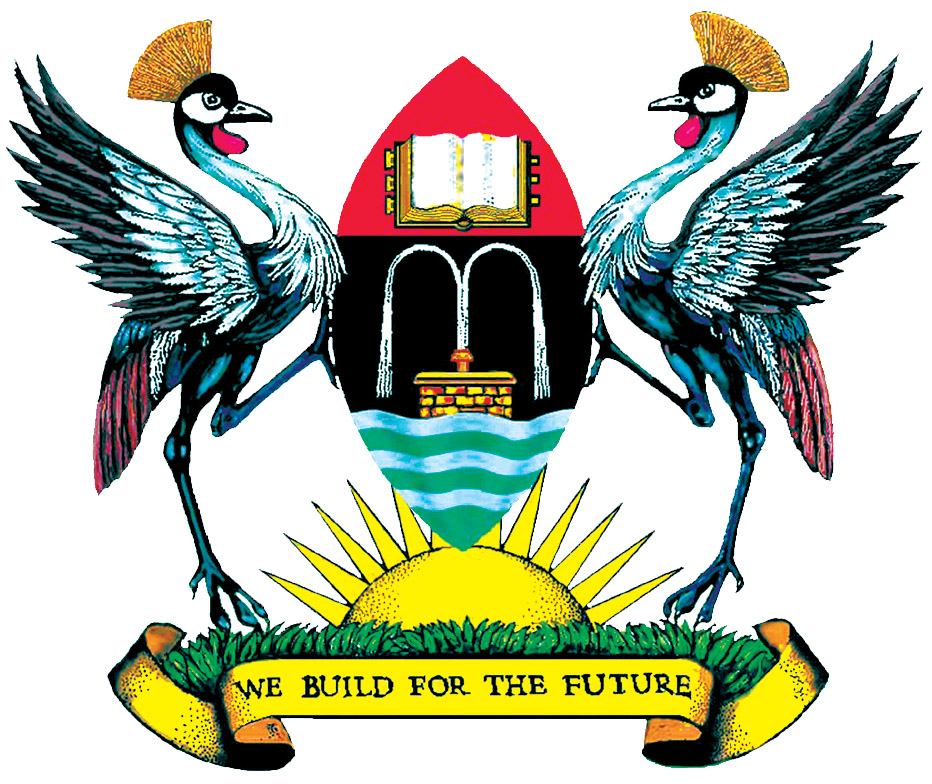


**DEPARTMENT OF HEALTH POLICY PLANNING AND MANAGEMENT,**

**MAKERERE UNIVERSITY SCHOOL OF PUBLIC HEALTH**

# Table of Contents

Table of Contents [2](#__RefHeading___Toc95382579)

Introduction [3](#__RefHeading___Toc95382580)

Section 1 – How to recognize danger signs in a very sick child [6](#__RefHeading___Toc95382581)

Section 2 – How to recognize ARI [10](#__RefHeading___Toc95382582)

What is ARI? [10](#__RefHeading___Toc95382583)

Section 3 – How to detect “Fast breathing” and “Chest Indrawing” [13](#__RefHeading___Toc95382584)

Demonstrating how to use the ARI timer [14](#__RefHeading___Toc95382585)

Measuring the breathing rate of a child [16](#__RefHeading___Toc95382586)

How to assess a child for chest indrawing [17](#__RefHeading___Toc95382587)

Section 4 – How to recognize fever in a sick child [19](#__RefHeading___Toc95382588)

Section 5 – How to diagnose malaria in a sick child [21](#__RefHeading___Toc95382589)

How can malaria be recognized in a sick child? [22](#__RefHeading___Toc95382590)

How is malaria prevented? [23](#__RefHeading___Toc95382591)

Section 6 - How to give treatment to a child with pneumonia or malaria [25](#__RefHeading___Toc95382592)

Treatment for pneumonia with amoxycillin [25](#__RefHeading___Toc95382593)

Amoxi dosage [25](#__RefHeading___Toc95382594)

Coartem (ACT) dosage [27](#__RefHeading___Toc95382595)

Tablet Feeding [28](#__RefHeading___Toc95382596)

Section 7 - How to use the diagnosis and management chart to guide treatment [31](#__RefHeading___Toc95382597)

Classification of Illness for the 4 month to 5 year old children [34](#__RefHeading___Toc95382598)

1) How to refer a child to a hospital or health centre [35](#__RefHeading___Toc95382599)

Section 8 – How to use the Follow-up and Referral Forms to keep records [38](#__RefHeading___Toc95382600)

Section 9 – How to manage supplies [42](#__RefHeading___Toc95382601)

Keeping medicines [42](#__RefHeading___Toc95382602)

Stock card and dispensing record [43](#__RefHeading___Toc95382603)

# Introduction

**What is the integrated management of malaria and pneumonia strategy?**

This is a strategy that takes malaria and pneumonia treatment to the home. It aims at reducing malaria and pneumonia morbidity and mortality in children. This will be achieved through:

- Providing high quality pre-packaged medicines at community level
- Training at least 2 CMDs for each village who will form part of the village health teams (VHT)
- Mobilizing communities particularly mothers to seek care early and to give appropriate home treatment
- Teaching both mothers and the CMDs to recognize and refer children with severe illness in time
- Counseling caretakers on the use of malaria preventive interventions particularly ITNs and IPT
- Improving quality of care at health facilities by ensuring that medicines are available and staff are competent and receptive

**Treating not only malaria but also pneumonia**

In African countries, children often fall sick from a number of different illnesses. Some of the common ones include malaria, acute respiratory infections (ARI), diarrhoea and measles. On a global scale, malaria is responsible for about one million children aged under five years dying each year, pneumonia is responsible for about two million and diarrhoea for about one and a half million. Other estimates indicate that of the approximately 10 and a half million children aged under five years that die annually, 20% are due to pneumonia, 17% due to diarrhoea, and 9% due to malaria (4% measles, 3% HIV/AIDS and 36% neonatal deaths). All these illnesses present with fever. If we are going to save more children from dying, it is important that treatment for these other common causes of fever, apart from malaria, are made available to those children that need them in time.

Fever in the first four months of life is a sign of possible bacterial infection which needs urgent attention in the health facility. In Africa, most episodes of fever in under-fives should be seen as potentially dangerous infections requiring careful monitoring or treatment.

This study is looking to find out whether community medicine distributors can safely diagnose pneumonia and malaria and give treatment. It is a community trial and will consist of two study arms, one called the intervention arm in which CMDs will carry out the routine HBMF strategy and use ARI timers to diagnose pneumonia, and a control arm in which CMDs will carry out the routine HBMF strategy only. The study will be carried out in Iganga – Mayuge Demographic Surveillance Site.

**Why is the Integrated Management of Pneumonia and Malaria necessary?**

Treatment of malaria and pneumonia at home is a common practice in Uganda. However, the way it is done is often incorrect or even dangerous. Access to professional health care is not as high as needed to treat the majority of febrile illnesses that children suffer. About 72% (2006 figures) of the population live within a distance of 5 km from a formal health facility (ranging from 7.1% in some rural districts to 100% in Kampala City). More than 80% of fever cases are first managed outside formal health facilities but in most cases wrong medicines are used. Even when correct medicines are used the doses are often incorrect or incomplete. Community Medicine Distributors have therefore been used in Uganda as a strategy to reach children with fever early and provide malaria treatment as close to the home as possible. Up till now, the malaria treatment used by CMDs was Homapak. The Ministry of Health has introduced a new malaria treatment called ACT (short for artemisinin-based combination therapy) using a brand of medicine called Coartem® (there are other types and brands of ACT). Even with this new and more effective malaria treatment, many children with fever are suffering from pneumonia, an illness which is potentially deadly if it is not treated in time. The treatment for pneumonia is an antibiotic, in this case you will be provided with Amoxicillin which is a penicillin type of antibiotic.

The integrated management of malaria and pneumonia strategy needs to be tested to make home treatment safer, more effective and easily accessible. In this training guide, CMDs will now learn how to identify fevers that are due to malaria and those that are due to pneumonia, in order to give better care for the sick child.

CMDs have an important role to play to support the Ministry of Health to achieve its target of treating 80% of children with fever within 24 hours of onset of symptoms by the year 2010.

**Is there evidence that community based management works?**

Yes, for example, in Ethiopia, educating mothers and giving them chloroquine reduced deaths from malaria in children under 5 years by 40%. In Burkina Faso, providing chloroquine at community level reduced the prevalence of severe forms of malaria by 50%. In several Asian countries, treating pneumonia at community level reduced child mortality by 50%. Here in Uganda, HBMF has been found to increase access to treatment within 24 hours and to reduce prevalence of severe anaemia.

**What is in this training guide?**

This guide is designed to serve as a teaching and reference tool for the trainers of the community medicine distributors (CMDs) participating in the intervention arm of the Home based management of malaria and pneumonia study. It is combining the training guidelines used for community case management of Acute Respiratory Infections (ARI) in Nepal with the HBMF Implementation guidelines for Uganda. These materials are intended to provide the CMDs the needed knowledge and skills to perform their roles in the WHO/TDR study. It has been organized into nine chapters and it is to be used together with the ARI and HBMF flipcharts as indicated in the various sections.

**How to use the training guide?**

The training is designed to be participatory with lots of activities and discussion. In order to teach the required skills, role plays, demonstrations, showing of videos and actual practice on real cases should be done. Also, many of the participants of the training may be illiterate so the trainer should use illustrations whenever possible. Throughout the training, the following steps should be taken:

- Ask CMDs questions about the key messages during the presentation of each session.
- At the end of each session have CMDs teach what they have learnt to each other in groups to demonstrate their knowledge and understanding.
- If any correction is needed, trainer should make the necessary corrections immediately.

The following pages should provide you with the step-by-step instructions on how to teach the manual. Each session is divided into the following sections:

- **OBJECTIVES**: This section is to remind the trainer what should be covered and understood by the end of each session. It is also recommended that the trainer use the objectives as a guide to measure knowledge and skills. Trainers should not move on to new sessions until most of the CMDs are able to perform the specific skills of the session.
- **MATERIALS**: This section is to remind the trainer what he or she needs to prepare or bring to the session.
- **BACKGROUND:** This section covers important information that the CMD needs to know in order to achieve the objectives of the session.
- **PROCEDURE**: This section describes in detail the steps that the CMD needs to know or follow in order to carry out an activity or task.
- **PRACTICE:** Exercises that can improve the skills of the CMD to carry out specific activities or tasks are outlined here. These include questions, drills or role plays. Evaluation of the CMDs' skills and knowledge should be continuous throughout the training.

The information in *italics* describes what the trainer should do. Trainers should encourage the CMDs that have appreciated a task or activity to assist the CMDs who are having some difficulty during the training. Trainers should attempt to conduct the sessions in both English and the local language and take advantage of those CMDs who can explain the materials in the local language to other CMDs. Important reminders are marked with an exclamation mark **!**. Important procedures to go through step-by-step with CMDs are marked with an .

**At the end of this training, the CMDs should be able to:**

- Identify signs of common childhood febrile illnesses
- Decide whether to refer a child to a health facility, or to help the family treat the child at home.
- For a child who is referred, advise the family to take the child to the nearest health facility as soon as possible.
- For a child who can be treated at home for malaria or pneumonia, help the family treat the illness at home.
- Counsel families to bring a child right away, if the child becomes sicker, and to return for scheduled follow-up visits.
- On a scheduled follow-up visit, identify the child’s progress and ensure good care at home; and, if the child does not improve, to refer the child to the health facility.

# Section 1 – How to recognize danger signs in a very sick child

Time: 2 hours

**OBJECTIVES**

By the end of this session the CMDs will be able to explain the danger signs in a very sick child aged 4 months to 5 years

**MATERIALS**

CMD Job Aid, HBMF flipchart, ARI flip chart.

**BACKGROUND**

Some sick children are so sick ill that they present with **danger signs** that indicate that the life of the child is threatened. A danger sign is a warning that the child is too sick and needs urgent treatment in a hospital or health centre. To help this child survive, you should **urgently** refer the child to the nearest hospital or health centre. In this section you will have the chance to know the danger signs and detect them.

The danger signs that you will come across in your work with children are listed in the box below:

**Box 1: Danger signs of a very sick child**

1) Convulsions or fits within the last two days or at present

2) Altered mental state (lethargy, drowsiness, unconsciousness or confusion)

3) Not able to drink or breastfeed

4) Vomiting everything or severe vomiting

5) Severe dehydration (sunken eyes, coated tongue, inability to drink)

6) Chest indrawing

7) Prostration (extreme weakness, unable to sit or stand)

8) Severe anaemia or “lack of blood” shown by pale lips or palms

9) Difficult or noisy breathing

**Convulsions**

During a convulsion, the child’s arms and legs stiffen. Sometimes the child stops breathing. The child may lose consciousness and for a short time cannot be awakened. When you ask about convulsions, use local words the caregiver understands to mean a convulsion from this illness. A convulsion during the child’s current illness is a danger sign. Refer a child with convulsions

**Altered mental state (lethargy, drowsiness, unconsciousness or confusion)**

Altered mental state means the child is drowsy most of the time when he should be awake and alert or continues to sleep when the mother talks to him or the mother claps her hands or starts to undress him or stares blankly and appears not to see. An unconscious child cannot be awakened by touch or pain. The child is very sick and needs to go to the health facility urgently to determine the cause and receive appropriate treatment. Refer a child who is very sleepy or unconscious.

**Not able to drink or breastfeed**

One of the first indications that a child is very sick is that it cannot drink or swallow. This is the case if the child has stopped drinking completely, rather than just reduced the amount that he or she drinks. Also, if a child vomits immediately after drinking, the child is considered “not able to drink.” Dehydration is a risk. Also, if the child is not able to drink or breastfeed anything, then the child will not be able to swallow the oral medicine you have in your medicine kit. Refer a child who is not able to drink, breastfeed or eat anything

**Vomiting everything**

If the child is vomiting, ask: “Is the child vomiting everything?” A child who is not able to hold anything down at all has the sign “vomits everything”. This child cannot hold down the medicine you have in your medicine kit. Ask the caregiver how often the child vomits. Is it every time the child swallows food or fluids, or only some times? A child who vomits several times but can hold down some fluids does not “vomit everything”. Refer a child who vomits everything.

**Severe dehydration (inability to drink, sunken eyes, coated tongue)**

A child who has been vomiting and failed to take enough fluid is likely to develop this sign. It signifies that the child does not have sufficient fluid in the body and is gradually drying up. The mother may alert you to this sign by telling you that the child is not able to drink and has been refusing fluids or feeds. The way to identify this sign is to look at the child carefully after exposing the face completely. A child with severe dehydration will have eyes that look as if they are sinking backwards into the head. These are called “sunken eyes”. If you are able to look into the child’s mouth you will notice that the mouth is dry and the tongue has a whitish coat. A child with severe dehydration needs to be treated at a hospital or health centre where the health worker will find a way to give the child fluids.

**Chest indrawing**

This is the case if the lower chest wall goes in when the child breathes in. When children have severe chest infection, they require greater effort to breathe. As a result, the chest wall moves in when the child breathes in. Chest indrawing is a sign of severe pneumonia. This child will need oxygen and appropriate medicine for severe pneumonia. Refer a child with chest indrawing.

**Prostration (extreme weakness, unable to sit or stand)**

A child that is very weak may not be able to sit or stand. If the child cannot do any of these actions as a result of an illness, then the child is said to have prostration. This means that the child needs urgent attention from a trained health worker and should be referred to the nearest hospital or health centre.

**Severe anaemia or “lack of blood” shown by pale lips or palms**

This sign occurs when a child does not have sufficient capacity in the blood to carry enough oxygen for his/her use. When this sign is present the child should be quickly taken to a hospital or health centre. The child with severe anaemia looks pale/whitish especially the lips and palms. The eyes look very white and if you examine the eyelids, you will find that they are white instead of pink. Refer a child with this sign.

**Noisy or laboured breathing**

Noisy breathing can have many different causes. Obstruction of the air passages of the nose, mouth, or throat may lead to noisy or laboured breathing. It is also a symptom of severe pneumonia. Listen if you can hear a grunting noise or stridor when the child breathes in or out, and whether the child seems to struggle to get air. Refer a child with this sign.

**PROCEDURE**

When a very sick child is brought to you do the following:

 Ask the caregiver what happened to the child

 Assess the child for any danger sign

 If the child has a danger sign, explain the seriousness of the sickness to the caregiver but do not create panic

 Explain to the caregiver that the child’s condition is too serious for you to handle and that better treatment can be provided at a hospital or health centre

 Ask the caregiver to take the child to the nearest hospital or health centre and give clear directions of how to get there

 Complete a referral note for the child

 Reassure the caregiver and firmly advise her/him to set off for the hospital or health centre as soon as possible

 Request the caregiver to let the CMD know the outcome of the referral when they return to the village

Important Reminder!

Refer a sick child who has one or more danger signs to the nearest hospital or health centre

**PRACTICE**

**Group work**

Divide into small groups of three or four participants. In each group discuss the danger signs by asking each one to describe two or three signs until you have discussed them all.

**Questions**

The trainer should randomly ask CMDs the following questions to evaluate the understanding of the danger signs in a sick child. Use these questions to create a discussion among the group.

Q1. What does a danger sign signify in a child who is sick?

Q2. How should you handle a child that is brought to you and is very sick?

Q3. When a child has a danger sign should you try to treat the child?

Q4. If you refer a child with a danger sign does it mean that you have failed to carry out your duty as a CMD?

Q5. Why is it important for the child with danger signs to be treated at a hospital or health centre?

Q6. Describe how you can identify:

- - Chest indrawing
  - Prostration
  - Convulsions
  - Not able to drink or breastfeed
  - Vomiting everything
  - Severe dehydration
  - Severe anaemia

# Section 2 – How to recognize ARI

Time: 4 hours

**OBJECTIVES**

By the end of the session CMDs should be able to explain and understand:

1. What is ARI

2. What is cough and cold

3. What is pneumonia

4. What may happen if pneumonia is not treated

5. What are the two age groupings for pneumonia assessment

**MATERIALS**

CMD flip charts, ARI video, IMCI video, Flip charts, Marker pens, Masking tape, TV and video.

**BACKGROUND**

## What is ARI?

ARI is a collective name of viral or bacterial infections of the respiratory system i.e. the chest. A variety of viruses and bacterial have been identified as causes for ARI in children. There are some risk factors that make some children more likely to develop ARI, specifically pneumonia. These factors include malnutrition, low birth weight, non-exclusive breastfeeding during the first four months of life, lack of measles immunization, indoor air pollution and crowding.

The common symptoms of ARI are sore throat, runny nose, nasal congestion, sneezing and cough; sometimes accompanied by 'pink eye', muscle aches, fatigue, malaise, headaches, muscle weakness, and/or loss of appetite. The forms of ARI are cold, cough and pneumonia. These are explained below

**Cold**

A child that has a runny or blocked nose is said to have a cold. Sometimes the child also has a cough. The parents or caregivers may treat a child with a cold or cough at home. A child with cold does not have fast breathing and the illness is usually mild.

**Cough**

A child with a cough has episodes of abrupt pushing out of air though the mouth that make a noticeable noise. Cough may occur on its own or with other symptoms such as cold, fever or breathing difficulty.

**Pneumonia**

A child with cough or cold or difficult breathing may have a more serious infection called pneumonia. Pneumonia is an infection of the lungs and a life threatening form of ARI. Pneumonia is a common cause of death in children and especially in young children. A child that has pneumonia needs urgent treatment with antibiotics otherwise the illness will get worse and the child may die.

It is difficult to tell the difference between pneumonia and a cough by the nature of the cough itself. Therefore when a child has a cough or cold, the caretaker should watch for signs of pneumonia. The signs that will show that the child has developed pneumonia are listed below:

**Box 2: Signs of pneumonia**

- Fever
- Difficulty in breathing such as (i) fast breathing or (ii) chest indrawing

**What are the signs of pneumonia?**

A child with pneumonia will have a fever or history of fever and difficulty in breathing. Difficulty in breathing will be recognized by any unusual pattern of breathing. Mothers may use such terms as “noisy”, “fast” or interrupted breathing to describe difficult breathing. Although difficulty in breathing occurs in children with pneumonia, it can also be detected in some children with cough or cold. That is why it is important to determine how serious the difficulty in breathing is in order to classify the ARI as mild e.g. cold or cough or severe e.g. pneumonia. To do this you have to assess the child for difficulty in breathing by looking for fast breathing and chest indrawing.

**Fast breathing**

This is present when the number of breaths made by a child in one minute is more than the normal number of breaths for the child’s age. This can be detected if the number of breaths are equal to or above a standard breathing rate for the child’s age. One breath includes both breathing in and breathing out. Fast breathing should be determined by counting the respiratory rate and not by the mother telling you. Fast breathing with chest indrawing is a sign of severe disease and requires urgent attention in a health facility.

**Chest indrawing**

Chest indrawing means that the lower chest wall goes **in** when the child breathes **in**. When children have severe chest infection, they require greater effort to breathe. As a result, the chest wall moves in. It is detected by observing the chest wall while the child is breathing. It is also one of the danger signs of a very sick child because it indicates that the chest infection is very serious and that the child is in danger of loosing their life.

There are ways to prevent ARIs and the CMD should know them so that a caregiver can be educated about them by the CMD. In the box below, is a list of preventive measures that can be taken.

**Box 3: Prevention of acute respiratory infections**

Keep young infants warm. Small and ill infants lose heat rapidly especially when wet. A young infant’s feet and hands should be kept warm always.

Avoid unnecessary exposure of all children to cold. To maintain the body temperature, children should always be well dressed.

Promotion of exclusive breastfeeding in children 0 to 6 months of age and continued breastfeeding for 2 years. Breast milk helps to protect against infections. Children who are breastfed are less likely to become seriously sick and die due to pneumonia.

Immunize all children, especially against measles and with the Penta vaccine, which protects against whooping cough and haemophilus influenzae. Immunizations strengthen the body to fight against infections, including pneumonia.

Avoid indoor air pollution. Children exposed to air pollution, such as smoke from indoor fires, are more likely to develop acute respiratory infection.

Avoid over crowding children while they sleep at night

**PRACTICE**

**Questions**

Following the lecture, the trainer should randomly ask CMDs the following questions to evaluate understanding of the differences between ARI, cough, cold, and pneumonia:

Q1. What part of the body do acute respiratory infections happen?

Q2. What is a cough?

Q3. What is a cold?

Q4. What is pneumonia?

Q5. Can a child with cough and cold develop pneumonia?

Q6. Can a child with pneumonia have a fever?

Q7. What may happen if a child with pneumonia is not treated?

Q8. What factors make children more likely to get ARI?

Q9. What advice can you give a mother to prevent her children from getting ARI?

Q10. When can you detect that pneumonia has become severe?

# Section 3 – How to detect “Fast breathing” and “Chest Indrawing”

Time: 4 hours (plus a visit to a health facility)

**OBJECTIVES**

By the end of the session, the CMDs will be able to:

1. Explain the fast breathing cutoff rates for both age groups

2. Realize that counting the breathing rate is necessary - estimating or guessing is not good enough

3. Operate the ARI timer

4. Count the number of breaths made by the trainer during one minute (when the CMDs are told when to start and stop counting)

5. Measure the breathing rate of a child using the timer and know the conditions necessary for measuring breathing rate (CMDs should practice counting on each other and on children displayed in the video)

6. Classify given cases as fast breathing or not fast breathing.

7. Distinguish between normal breathing and chest indrawing

**MATERIALS**

CMD flipchart, ARI timer, and 5-10 children less than 5 years of age, TV & video if possible and if electricity is available. Flip Charts, Marker Pens

**BACKGROUND**

A child with difficulty in breathing as a result of an ARI or any other cause, will have signs of fast breathing or chest indrawing. As explained in the previous section, a child without a chest infection should have a normal breathing rate and his/her chest should make normal movements.

**How is fast breathing measured?**

Fast breathing is measured according to the age of the child as follows (Box 4):

**Box 4: The number of breaths in a child that are detected as fast breathing**

• For a child 4 months up to 12 months of age fast breathing is 50 or more breaths per minute.

• For a child 12 months to 5 years of age fast breathing is 40 or more breaths per minute

The number of breaths are counted when the child is settled. The CMD has to become proficient in counting the breathing rate. The rate comes down with age because of the larger chest cavity in adults that can take in more air.

**How is chest indrawing detected?**

When a child has to use much effort to breathe, the lower chest wall moves in as the child breathes in. This movement is unusual and is called “chest indrawing”. To identify this sign of breathing difficulty, the CMD has to watch the chest wall as the child is breathing.

**PROCEDURE**

**Demonstrating abnormal breathing rate**

 Select one participant to stand in front of you by about four feet

 Ask the participant to observe your chest and the way you are breathing to decide if you are breathing normally or abnormally. After each example ask the CMD to describe what he/she saw and whether normal or abnormal (as you do the breathing examples turn your head away from the person in front of you so that you do not exhale directly into their face) and compare the response with the rest of the class in order to get a discussion going:

 Vary your breathing pattern as follows:

a) Breathe normally for about 20 seconds

b) Take deeper breaths than usual but maintain a normal rate for the same duration as above

c) Increase the rate of your breathing but maintain a normal depth

d) Increase the rate of your breathing and increase the depth

*You should count your own breathing rate as well to confirm the responses from participants. Use the different answers to illustrate the need for a standard method of counting breathing rates more accurately.*

**Demonstrating how to count breaths in one minute**

1. Select one participant with a watch to keep time and another to stand in front of you by about four feet.

2. Explain to the participants that you will breathe and that he/she should count the number of breaths you take in one minute. The other participant with the watch will count one minute by telling the other when to start and stop counting.

3. The other CMDs should participate by confirming the number of breaths.

4. As above vary the pattern as follows:

a) Breathe normally

b) Take deeper breaths than usual but maintain a normal rate

c) Increase the rate of your breathing but maintain a normal depth

d) Increase the rate of your breathing and increase the depth

*You should count your own breathing rate as well. Use the similar answers to achieve agreement in the number of breaths in one minute. Show that by having a define time period to count the breaths a standard method has developed.*

*Do not proceed until most CMDs can count your breathing rate accurately. Repeat until this is achieved.*

## Demonstrating how to use the ARI timer

Explain to participants that when they are assessing a sick child, they will not have someone to monitor the time for them but will have to do so themselves. It is possible to use a watch or clock but it will mean that the CMD will not be able to properly watch the child’s breathing and can miss some of the breaths. To help the CMD to monitor the time, an ARI timer can be used. Show the actual timer, but do not distribute them yet. Explain further that the timer makes a sound after half a minute and a different sound after one minute. Since it makes a sound, you do not have to look at the timer while you are using it, you can concentrate on counting the breathing rate of the child. Show the following:

1. To start the timer, you press the center circle. **(Do this).** The timer makes a "beep" to tell you that it has started. If you listen carefully you will hear a ticking noise. This tells you the timer is working.

2. At half a minute the timer will make a "beep" sound. **(Wait for this sound)**. Then it continues ticking to the end of the minute when it makes a "beep-beep" sound. **(Wait for this sound)**.

3. Now the timer stops by itself and is ready to be used again. You should **NOT** stop the timer at the end of the minute.

*Demonstrate how the timer works a second time*

If you start the timer and then want to cancel your count, you can stop the timer by pressing the center circle again. **(Demonstrate this)**. If you start the timer again, it starts at the beginning of the minute - not part way through.

**PRACTICE**

*Hand out the timers. Ask every CMD to:*

- start the timer
- listen to the ticking
- listen to the half minute sound
- listen to the minute sound
- start the timer again and listen to the ticking sound
- stop the timer part way through the minute

*Allow the CMDs about three or four minutes to explore the use of the timer.*

Let them ask questions about the timer.

**Using the timer to count the breathing rate**

*Explain to the CMDs:*

- This time you will have to time the minute for yourselves as well as doing the counting.
- To divide themselves in to groups of two and to take turns counting their partner’s breathing rate. The partner should count his/her own breathing rate to compare.

*Remind the CMDs not to count the tick of the timer.*

Start timing and counting as soon as you are ready **after** you get the signal from the trainer.

*Ask the CMDs:*

- Please tell your partner the number of breaths that you counted during the minute.
- Check the answers and give feedback. Continue the above steps until each pair gives answers which are within 2 breaths of each other’s count**.**

## Measuring the breathing rate of a child

These exercises can be done at a health centre with children aged under five years or using the video

*Explain to the CMDs:*

To decide whether a breathing rate is normal or fast one has to count the child’s breathing rate and compare it with the normal rate for the child’s age. To do this, practice the following:

- Be sure the child is calm. If the child is crying or screaming, wait until the child calms down before you start to measure breathing rate
- You should first decide where to observe the child's breathing. Look wherever the breathing is obvious. This may be the child's chest or abdomen. The location does not matter, provided that you can clearly see the breathing movements.
- You should ask the parent to lift the child's shirt so that you can see the child's chest and abdomen. Do not start the timer or begin counting until you are focused on the child. You must wait until the child is calm before starting the count.
- Stand where you can see the child’s breathing easily. Look at the child’s lower part of the chest. This is the part where the last rib can be seen.
- Push the Start/Stop button once. A short "beep" sound will be heard to show that the timer is starting. Actual counting of the respiratory rate must begin as soon this initial beep is heard. Audible clicks will sound every second so that you can be sure that timing is in progress while you continue to observe the patient.
- The timer will give another short beep at the 30 second (half a minute) point of the timing cycle, and two short beeps at the end of 60 seconds (one minute) when it will automatically shut off. Stop counting when the two short beeps are heard.
- Record the rate and repeat the measurement. It is important to get two measures to be sure that the results are accurate.
- Take the second measurement if it is within 2 breaths of the first. If it is very different repeat the count until you get two counts that are within 2 breaths.
- Ask the caregiver for the age of the child and compare your count with the normal count for the child’s age.
- If the child moves or cries during the counting, the count is invalid and must be retaken. A high temperature can also cause the breathing rate to be higher than normal.
- The child may be sleeping or breast feeding during the count. This is alright.

*Ask the CMDs:*

- To divide themselves into small groups of three or four, with one child for each group.
- Within each group, the CMDs should count the number of breaths per minute for the child, repeating the steps given in the Demonstrating how to count breaths in one minute section. The trainers should move around from group to group to evaluate if the CMDs are counting properly.

**Do not move on to the next section until all CMDs are able to count the breathing rate of the child to an accuracy of + or - 2 breaths per minute.** If only one or two CMDs are having difficulty, you should move on to the next section but provide extra teaching during break time or at the end of the day.

**How to decide whether a given breathing rate is fast**

*Explain to the CMDs:*

- The number of breaths in one minute is called the breathing rate (or respiratory rate but for standardization we shall use breathing rate for this study).
- The breathing rate for infants (children aged between 4 months and up to 12 months) is normally faster than for older children (12 months to 5 years of age). Young children have faster breathing rates than adults.

**Interpretation of the results**

+ For a child aged 4 months up to 12 months, fast breathing is 50 or more breaths per minute

+ For a child 12 months up to 5 year olds, fast breathing is 40 or more breaths per minute.

*Explain to the CMDs:*

I am going to tell you about several children. For each child I want you to tell me how you would classify the child. You can classify the child as "Fast" or "Normal". They can refer to their notes or job aid.

*Say:*

The first child is 6 months old and his breathing rate is 40 breaths per minute. Is the child’s breathing rate normal or fast?

Repeat the steps above for the following cases:

| **Age of child** | **Breathing rate** | **Correct response** |
| --- | --- | --- |
| 4 months | 53 | Fast |
| 11 months | 51 | Fast |
| 3 years | 36 | Normal |
| 8 months | 58 | Fast |
| 2 years | 38 | Normal |
| 1 year | 39 | Normal |

*Add other examples if you feel this is necessary to achieve full accuracy.*

## How to assess a child for chest indrawing

*Explain to the CMDs:*

- When assessing a child for indrawing you should not disturb the child so that he/she stays calm.
- The mother (and not you) should lift the child’s clothing to expose the chest.
- Observe the movements of the lower chest wall. To detect chest indrawing the whole area of the lower part of the chest moves in, rather than just the spaces between the ribs. The inward movement happens whilst the child breathes in and it happens every time that the child breathes in.
- The chest indrawing occurs even while the child is calm.
- If chest indrawing cannot be seen clearly, the child should be moved so that he/she is lying flat in the mother’s lap.
- If chest indrawing is still not clearly visible, the child should be classed as not having chest indrawing.

**Interpretation of results**

The presence of chest indrawing means the child has severe pneumonia. Chest in drawing is a danger sign. A child with chest indrawing should be referred to the nearest hospital or health facility as soon as possible.

**Video of Fast breathing and Chest indrawing**

If a video machine is available and electricity, the video on fast breathing and chest indrawing should be shown to the participants. If it is not possible to show the video, real cases of chest indrawing should be sought in the health facility. If neither are possible then arrange for another occasion when the CMDs can practically review an example of chest indrawing.

# Section 4 – How to recognize fever in a sick child

Time: 2 hours

**OBJECTIVES**

The objectives of the training are to orient CMDs to recognition of fever

**MATERIALS**

Notebooks, pens, a flip chart and markers

**BACKGROUND**

Fever can be a symptom or a sign. When a caregiver tells you that the child has fever, that is a symptom whereas when you as a CMD touch the child and find out that the forehead is warmer than normal, you have detected fever as a sign. Fever is a common symptom of sickness in a child. The commonest causes of fever in children aged under five years are malaria, acute respiratory infections (ARI), diarrhoeal diseases and other viral infections such as measles. Fever is therefore not a specific symptom or sign of any of these sicknesses. That is why it is important to assess a child to try to find out the specific cause of the fever. It is common to think that all fevers in children are caused by malaria but this is not always the case. It is for this reason that this study is taking place to try to address ARI, the other common cause of fever in children.

A child with fever may also have other complaints and signs. These include:

- Vomiting
- Refusal to feed
- Excessive sweating
- Lethargy (lack of interest, not playing as usual)
- General body weakness

If a child with fever is not managed properly the fever may get worse causing the body temperature to rise very high. Very high fevers can cause the child to have convulsions (called febrile convulsions) or to become very irritable or restless. A child with fever and any of the danger signs is critically sick and needs to be referred to the nearest hospital or health centre as soon as possible.

**PROCEDURE**

**Recognition of a child with fever**

Fever simply means hotness of the body as a result of it having an abnormally high core temperature. A CMD should presume that a child has fever if:

 The caregiver says that the child has or had a fever. We should believe what the caregiver says even if the body of the patient is not hot because sometimes the fever can reduce then return again a few hours later. Confirm with the caregiver how she/he found out that the child was hot.

 The body of the child is hot on touching the forehead with the back of your hands (not the palm side). Sometimes this method may give a wrong finding especially in cold weather when you palms or the forehead of the child may be cold yet the internal body is abnormally hot.

# Section 5 – How to diagnose malaria in a sick child

Time: 4 hours

**OBJECTIVES**

The objectives of the training are:

1. To orient CMDs to recognition of malaria symptoms
2. To remind the CMDs about their roles in HBMF strategy implementation

**MATERIALS**

Notebooks, pens, a flip chart and markers, CMD Registers, HMBF flip chart, malaria transmission cycle poster.

**BACKGROUND**

**Information on malaria**

Malaria is the most common cause of illness and death among children under 5 years of age in Uganda. Most of the visits and admissions to health facilities are due to malaria. Malaria can be prevented and treated.

**Transmission of Malaria**

Transmission of malaria occurs from person to person through the female *Anopheles* mosquito. The mosquito carries the malaria parasite, called *Plasmodium falciparum*, from an infected person to another person while it is feeding on human blood. The mosquito needs the blood meal to develop its eggs and breed another generation of mosquitoes. A person gets malaria when a mosquito that is carrying malaria parasites bites them. Use the malaria transmission cycle poster to describe the steps that you need to know about how malaria is carried from one person to another.

**Steps of malaria transmission**

**Step One**: A mosquito carrying malaria parasites will land on a person during the night. In the process of feeding on that person, it injects its saliva and sucks in the blood. The malaria parasites are in the saliva and in this way are transmitted from the mosquito to the person.

**Step Two**: Once in the human blood, the malaria parasites develop and multiply. As the malaria parasites multiply they become so many, causing the person to begin to feel sick. From the time of the infected mosquito bite, it takes from 7 to 14 days for the person to feel sick. Once this happens the person has malaria disease (uncomplicated malaria).

**Step Three**: Malaria requires quick treatment that is given early enough to avoid the child (or person) getting worse. Some children, if not treated or not treated with an effective malaria treatment will develop danger signs and progress to severe malaria. Severe malaria requires urgent treatment at a health facility by a trained health worker.

**Step Four**: The female *Anopheles* mosquito after mating with a male mosquito lays between 30 and 200 eggs every 2-3 days. The eggs are laid at the edges of collections of stagnant water especially water that is not very muddy or dirty. The eggs develop into the mature adults in about 2 to 7 days. The female adult *Anopheles* mosquito prefers to feed indoors on human blood to provide nourishment for its new eggs.

**Step Five**: Some people carry malaria parasites but are not sick with the disease. When a mosquito feeds on such a person, it sucks in blood with the malaria parasites. After a good feed, the mosquito tends to rest on the wall to allow digestion to take place before flying away. The malaria parasites undergo another stage of development inside the mosquito lasting about two weeks before they reach the salivary glands of the mosquito ready to be injected into the next person that the mosquito feeds on. The cycle then starts again. In this way, one mosquito during its life span of about two weeks can lay on average over 300 eggs and transmit malaria on average once.

**Important facts about malaria mosquitoes and malaria transmission**

- Malaria can occur throughout the year, but it is most common during, or just after, the rainy season.
- Many mosquitoes come out at night and are bothersome, but not all mosquitoes transmit malaria.
- Malaria mosquitoes usually feed late at night (10 pm) to early morning (4 am). They usually enter the house in the late evening (from about 5 pm to about 10pm)
- Malaria mosquitoes can breed even in small amounts of water such as a foot print in the rainy season. This is why it is difficult to control their breeding especially when the climatic conditions are conducive for the mosquitoes to breed and the malaria parasites to develop and mature in the mosquito.

**Who are at highest risk of illness and death from malaria?**

- Children aged under five years of age
- Pregnant women
- People with chronic illnesses such as HIV/AIDS, and sickle cell

These groups of people do not have enough immunity to prevent themselves from getting frequent attacks of malaria or the severe form of malaria. It is important to know who these people are since they need special protection from malaria. Families and communities should take special care to ensure that these people do not get infected with malaria.

## How can malaria be recognized in a sick child?

- The most common symptom of malaria is fever (hot body) or history of fever. The caregiver will give this information. If there is no fever (hot body) or history of fever, then it is not likely to be malaria.
- Malaria can also cause vomiting, diarrhea, cough and irritability
- Malaria can occur at the same time with other causes of fever such as ARI. In this case, the child will have the symptoms of both or more diseases.

The features above describe simple malaria in a child. However malaria can also be severe causing the child to become very ill and even die. A child with severe malaria will have one of more danger signs and should be referred immediately to the hospital or health centre.

**How is malaria diagnosed?**

Every fever is not caused by malaria. As you can see the symptoms of malaria are similar to those of other childhood diseases such as ARI and diarrhoeal disease therefore if one depends on the symptoms there is a chance of making a mistake. To improve the chances of correctly detecting malaria one can look for the parasites in the blood of the sick child. One method of doing this is to do a blood smear and checking it under a microscope. This can be done in a laboratory by a trained laboratory technician. Still, for treatment in community, we base the malaria diagnosis on the presence of fever.

## How is malaria prevented?

Some ways of preventing malaria are listed below.

*You can refer to the malaria transmission cycle to explain*

**The methods of preventing against malaria**

- Sleeping under insecticide treated bed-nets (ITNs) prevents the mosquito from getting a blood meal and when the mosquito lands on the net some of the insecticide enters into the mosquito causing it to die shortly thereafter. ITNs have been shown to be safe and are very effective when used properly every day.
- Carrying out indoor residual spraying (IRS) causes the mosquito to come in contact with the insecticide on the wall which kills the mosquito. This is also a very effective method and uses special insecticides that are sprayed inside the house. Both ITNs and IRS also have a repellant effect on some mosquitoes by discouraging them from entering the house or once they enter leaving without having a blood meal.
- Taking malaria medicine at regular intervals to reduce the number of malaria parasites in the human blood. This can be used for intermittent preventive treatment (IPT) and is commonly used for pregnant women. In special groups such as sicklers or tourists to malaria endemic countries, chemoprophylaxis is practiced. A malaria medicine is given on a continuous and regular basis.
- Use of mosquito sprays and coils that either kill or repel mosquitoes. These are not as effective as ITNs or IRS.
- Using mosquito repellants. Repellants are special chemicals that can be applied on the skin to prevent mosquitoes from coming in contact with the person. These tend to be expensive and have to be used every time one is likely to be exposed to mosquitoes.
- Wearing clothes that cover your body, especially in the night. This method is also not very effective.
- Screening all house windows with nets with small holes that prevent the mosquito from getting into the house through the windows. This is quite expensive and more feasible in urban areas. To be effective, one has to develop the habit of closing windows and doors after 5 pm and early in the morning.
- Larviciding by killing the mosquito larvae that breed in water. This method is of limited effectiveness in Uganda because our type of malaria mosquito, *Anopheles gambiae*, can breed in any small fresh collections of water. However larviciding can be used in areas next to large well-defined collections of stagnant water.
- Filling in or draining places where water stagnates and mosquitoes breed. This method is not very effective in Uganda because our type of malaria mosquito can breed in small fresh collections of water. However it is still good practice to prevent mosquitoes from breeding close to the house in obvious places and at construction sites where numerous sources of stagnant water are likely.

**How is malaria treated?**

The treatment for malaria that is recommended by the Ministry of Health is the medicine called Artemether/lumefantrine (also called Coartem). This medicine is an artemisinin-based combination therapy (called ACT in short). The Ministry of Health provides Coartem through health facilities and community medicine distributors. As a CMD it is your role to give this medicine to children aged between four months and under five years with fever and in a safe way. In this study, we shall give you the training to diagnose malaria so that you can give the medicine to those children that have malaria.

Children who have severe malaria have to be treated in a hospital or health centre with a more powerful medicine.

**PRACTICE**

The trainer should ask the CMDs questions to determine their understanding of the contents of this section.

Q1. What is responsible for transmitting malaria?

Q2. Describe the way in which malaria is transmitted.

Q3. Who are at high risk of getting malaria?

Q4. What are the common symptoms of malaria? Are they specific only to malaria?

Q5. What methods can be used to diagnose malaria?

Q6. Give three ways of preventing malaria.

Q7. What medicine is now used to treat malaria?

# Section 6 - How to give treatment to a child with pneumonia or malaria

Time: 4 hours

**OBJECTIVES**

By the end of the session the CMDs will be able to explain:

1. The kind of medicine to give a child with pneumonia or malaria
2. The treatment dosages of Amoxil for each age group as treatment for pneumonia
3. The treatment dosages of Coartem for each age group as treatment for malaria
4. How to teach mothers the dosages and to prepare and give tablets

**MATERIALS**

CMD ARI flipchart, HBMF flipchart, spoons, tablets, clean water, bowls and treatment cards

**BACKGROUND**

To make it easier to give the right dose, the medicines have been divided into pre-packs containing one dose for a specific age group. It is therefore important that you learn the doses and how they have been packed and colour coded by age group.

**PROCEDURE for pneumonia treatment**

## Treatment for pneumonia with amoxycillin

*Explain the CMDs:*

If a 4 month to 5 year old has pneumonia you should treat the child with Amoxi tablets. Amoxi is an antibiotic. Show the tablets to the CMDs and let them pass the tablets around.

Have the trainees turn to appropriate page of CMD ARI flipchart and have the trainer or a CMD read the page to the rest of the group. Explain the text to the rest of the group.

Each of these boxes is supposed to represent a day. There are 3 days. So the mother is supposed to feed the 4 month to 1 year old 1 tablet in the morning and 1 tablet in the evening for 3 days. Repeat the instructions for the older age groups.

*Explain to them the two different age groups for Amoxi dosages.*

## Amoxi dosage

| **Age** | **Day 1** | **Day 2** | **Day 3** | **Colour Code** |
| --- | --- | --- | --- | --- |
| From 4 months to 12 months | 1 tablet twice a day | 1 tablet twice a day | 1 tablet twice a day | Pink |
| From 12 months to 36 months (1-3 years) | 2 tablets twice a day | 2 tablets twice a day | 2 tablets twice a day | Green |
| From 36 months to 59 months (3-5 years) | 3 tablets twice a day | 3 tablets twice a day | 3 tablets twice a day | Red |

**Note**

- Even if the child improves after 1 or 2 days, the mother must complete the 3-day course of Amoxi tablets.
- Home care should be given until the child has completely recovered.
- A few people are known to react to this type of medicine, so if the caregiver tells you that her child reacts to the medicine (called adverse drug reaction or locally as bad medicine reaction), avoid giving the medicine and refer instead.

**PRACTICE**

**Questions**

*Ask the CMDs:*

Q1. What is the Amoxi pediatric tablet dosage for a 4 month to 1 year old?

Q2. What is the Amoxi pediatric tablet dosage for a 1 to 3 year old?

Q3. What is the tablet dosage for a 3 to 5 year old?

Q4. What else should be taught to the mother?

**Drill**

The trainer should keep asking the CMDs the dosages for different ages to see if they understand. Drill the CMDs for about 5 minutes. The CMDs break into pairs and teach the dosages to each other. Trainer should move from pair to pair to evaluate the teaching. Make any necessary corrections.

**Role play**

Select a volunteer from the participants to perform a role play. In this exercise, you will learn how to teach the dosages to the caregiver using the CMD flipchart. The trainer will be the "CMD" and a CMD will play the role of the "mother". The trainer should ask the child's age since it is needed for dosage purposes. Explain the cause of the child’s illness, the nature of the treatment and the right dosage. Counsel the mother about prevention of ARI and comfort her that her child will recover. Tell her to return to you if the child does not improve or go to the hospital or health centre if the child gets worse.

After the play, the trainer should discuss with the group the important aspects of the exchange between a caregiver and CMD. If the class still has queries that you need to address, repeat the play. Then get two other volunteers to repeat the play and ask the class to criticize their performance giving the good and bad points.

**PROCEDURE for malaria treatment**

**Treatment for malaria with Coartem**

*Explain the CMDs:*

- The medicine for treating sick children was changed from a pack containing Chloroquine + Sulfadoxine/Pyrimethamine (Homapak™) to one containing Artemether/Lumefantrine (Coartem®). Coartem® is a type of malaria treatment called ACT. Show the tablets to the CMDs. Let them pass the tablets around.
- If a 4 month to 5 year old has fever and a positive RDT the child can be treated by you with ACT tablets.
- An ACT such as Coartem® is a safe and effective medicine for treating malaria.
- The age range of children to be treated under HBMF is now from 4 months up to 5 years.
- The pack for children aged 4 months up to 3 years is YELLOW and that for children aged 3 years up to 5 years is BLUE.

*Ask the CMDs to turn to appropriate page of CMD Job Aid and explain to them the two different age groups for dosages.*

Always make sure that a sick child receives the correct pack for age as shown in the table.

## Coartem (ACT) dosage

| **Age** | **Day 1** | **Day 2** | **Day 3** | **Colour Code** |
| --- | --- | --- | --- | --- |
| From 4 months up to 3 years | 1 tablet  twice a day | 1 tablet  twice a day | 1 tablet  twice a day | Yellow |
| From 3 years up to 5 years | 2 tablets  twice a day | 2 tablets  twice a day | 2 tablets  twice a day | Blue |

It is important to ensure that the full course of Coartem® is taken even when the child feels better before completing the course

**Note**

- Even if the child improves after 1 or 2 days, the mother must complete the 3 day course of Coartem® tablets.
- If the child has cold or cough the caregiver should given home care in addition to the medicine
- Adverse drug reactions (i.e. bad medicine reactions) to this medicine are very rare but ask the mother to return to you if the child does not improve or to go to hospital or a health centre if the child gets worse.
- A few children may have fever and concurrent infections with both pneumonia and malaria. In such a case, the child will need paracetamol for the fever, an antibiotic for the pneumonia and a malaria treatment for the malaria.
- Ideally Coartem® should be given with some milk or food that contains some fat/oil. When this cannot be done, the medicine should still be given anyway.

**PRACTICE**

**Questions**

*Ask the CMDs:*

Q1. What is the new treatment for malaria that the Ministry of Health recommends?

Q2. What is the Coartem® tablet dosage for a 4 month to 3 year old?

Q3. What is the Coartem® tablet dosage for a 3 to 5 year old?

Q4. What else should be taught to the mother?

Q5. Why is Homapak no longer used?

Q6. How do you determine if a child should get Coartem®?

**Drill**

The trainer should keep asking the CMDs the dosages for different ages to see if they understand. Drill the CMDs for about 5 minutes.

The CMDs break into pairs and teach the dosages to each other. Trainer should move from pair to pair to evaluate the teaching. Make any necessary corrections.

**Role play**

Select a volunteer from the participants to perform a role play. In this exercise, you will learn how to teach the dosages to the caregiver using the CMD job aid. The trainer will be the "CMD" and a CMD will play the role of the "mother". The trainer should ask the child's age since it is needed for dosage purposes. Explain the cause of the child’s illness, the nature of the treatment and the right dosage. Counsel the mother about prevention of malaria and comfort her that her child will recover. Tell her to return to you if the child does not improve or go to the hospital or health centre if the child gets worse.

After the play, the trainer should discuss with the group the important aspects of the exchange between a caregiver and CMD. If the class still has queries that you need to address, repeat the play. Then get two other volunteers to repeat the play and ask the class to criticize their performance giving the good and bad points.

**PROCEDURE for tablet feeding**

## Tablet Feeding

Turn to appropriate age of CMD flipchart and ask CMD to read and explain the pictures.

*Explain to the CMDs:*

- How to feed tablets to a child? Discuss the different ways the tablets can be given also depending on the age of the child. It is important to clarify with the class how to arrange for clean drinking water to use during their work in the community.
- The importance of the CMD giving the first dose.
- The importance of using clean and safe drinking water to mix the crushed tablets and give the first dose. Discuss possible sources of clean water for this purpose

**Trainer should demonstrate how to crush and mix tablets.**

Break the CMDs into groups of four persons and give them each some tablets, water, a spoon and a cup/metal bowl. Ask each member of the group to practice crushing and mixing the tablets with water. The trainer should move from group to group to observe that they are doing it correctly, i.e. the CMDs should not use too much water. When the groups have finished crushing and mixing the tablets, discuss with them their experiences including experiences from their work in the community.

**PROCEDURE for counseling a caregiver**

*Explain to the CMDs:*

We shall now combine all that we have learnt to know how to handle a caregiver to ensure that he/she understands what is wrong with the child and how to give the medicine correctly. To be systematic you should do the following:

 Explain to the caregiver the cause of the child’s illness

 Explain the treatment that you are going to give and the dose for her child.

 Explain to the caregiver to give the doses every day for 3 days and that even if the child seems better, the complete treatment should be given.

 Show the mother how to crush the tablets. Emphasize the need to wash hands before and after giving the tablets.

 Show the mother how to mix the crushed tablets with a small amount of water. Emphasize that clean drinking water should be used.

 Explain to the mother to give the dose again if the child vomits within half an hour of taking the last dose

 Check that the mother has understood by asking questions about what she will do.

 Prepare the tablets and give the child the first dose.

 Observe the child for the next 30 minutes to see if the child vomits the medicine. If the child vomits the medicine within 30 minutes, repeat the dose.

**PRACTICE**

**Role play**

Get a volunteer and practice the steps about. The trainer should act as the CMD and the CMD as the caregiver. Counsel the caregiver as you would do in the community. Show how to crush the tablet/s and mix with water. At the end of the role play and ask the rest of the class their opinion of the role play.

If there is time left at the end of the session, ask two more CMDs to role play. Repeat with another pair until the class is happy with the performance.

**PROCEDURE for detecting adverse drug reactions**

*Explain to the CMDs*

 That medicines can produce reactions in people which can make the patient to feel more uncomfortable. These reactions are called adverse drug reactions or locally bad medicine reactions.

 The reactions may be mild or severe; they can be transient i.e. disappear when the medicine is stopped or in rare cases they can be permanent.

 Examples of some adverse drug reactions include the following itchiness, nausea, vomiting, dizziness, fatigue and excessive sleepiness. Some reactions overlap with the symptoms of febrile illnesses

 The challenge sometimes is to differentiate the reactions to the medicine from the disease that the patient has taken the medicine to treat.

 As a CMD it is important that you make caregivers aware that they should report to you any changes in the patient that indicate that the patient is getting worse or more uncomfortable e.g. a skin rash. When such a report is made to you, the CMD should record the bad medicine reaction in the CMD register and study follow up form.

**PRACTICE**

**Questions**

Q1. What are adverse drug reactions? How would you explain them to a caregiver in

the local language?

Q2. What adverse drug reactions do you know of? How can you differentiate them

from the symptoms of malaria?

Q3. Explain how you would handle a 2 year old child with excessive vomiting that

started on the second day of treatment with a medicine such as Coartem?

# Section 7 - How to use the diagnosis and management chart to guide treatment

Time: 4 hours

**OBJECTIVES**

By the end of the session, CMDs will be able to:

1. Know which questions to ask the mother
2. Know what to look and feel for
3. Know how to use Amoxi to treat children with pneumonia
4. Know how to use Coartem to treat children with malaria

**MATERIALS**

Fever diagnosis and management chart, flip chart and markers.

**BACKGROUND**

To handle a child with fever and give the right treatment it is important that one should be systematic by following a step by step process. The principle that the CMD will use is based on “ASK, LOOK and FEEL”. What this means is that the CMD should first ask the caregiver a series of questions to understand the symptoms that the child has then the CMD should look at the child and make any observations such as the breathing rate and finally the CMD can feel the child to carry out things like temperature measurement. After these assessments the CMD will be able to decide what actions to take including giving the appropriate treatment according to the fever diagnosis chart.

The CMD should be careful to give the right medicine and the right dose. The caregiver should be explained the reason for giving the treatment and shown how to take the medicine. It is important to take the time to explain and make the caregiver understand the medicine and its dose because this will encourage the caregiver to give the complete dose. In this way the child is more likely to make a full recovery. Whenever possible the CMD should administer the first dose of the medicines showing the caregiver the way to do so. The CMD can then counsel the caregiver on ways to prevent the child’s illness, that is if it is malaria or an ARI.

It is the role of the CMD to follow up the sick child to find out if the child has made a complete recovery or become worse. If the child is worse, the CMD should complete a referral note and ask the caregiver to take the child to the nearest hospital or health centre.

**PROCEDURE**

Make sure that each CMD has a copy of the fever diagnosis and management chart. Take them through the chart carefully and referring to any other sections of this manual and job aids that have been used.

**ASK**

**Ask the name and age of the child?** You need the child's age for several reasons. You need to know the child’s age for classifying the breathing rate (4 months-12 months or 12 months-5 years), deciding on treatment dose of Amoxi and Coartem. If the mother is not sure of the age you can ask if the birth happened before or after certain important events, such as elections, holidays etc. If the child is below 4 months and above 5 years, you should refer the child to the nearest hospital or health centre. A CMD should not handle these patients because they do not have the right medicines to give.

**Ask the caregiver for the child’s main health problems?** Does the child have a history of fever? Cough? Cold?

The next step is to find out if the child has any danger signs. Start by asking for symptoms as follows:

**Has the child had convulsions?** You should ask the mother if the child has had a convulsion whereby the child’s arms and legs stiffen. Sometimes the child stops breathing. The child may lose consciousness and for a short time cannot be awakened. When you ask about convulsions, use local words the caregiver understands to mean a convulsion from this illness.

**Is the child vomiting everything?** If the child is vomiting, ask: “Is the child vomiting everything?” A child who is not able to hold anything down at all has the sign “vomits everything”. Ask the caregiver how often the child vomits. Is it every time the child swallows food or fluids, or only some times? A child who vomits several times but can hold down some fluids does not “vomit everything”.

**Is the 4 month to 5 year old able to drink or breastfeed?** You should also ask the mother if the child has stopped drinking completely, rather than just reduced the amount that he or she drinks. Also, if a child vomits immediately after drinking, the child is considered “not able to drink.” During the assessment, you should also ask the mother to offer the child something to drink in front of you to see for yourself if the child is really able to drink or not.

**Is the child abnormally sleepy or difficult to wake?** Remember, "abnormally sleepy or difficult to wake" means the child is drowsy most of the time when he should be awake and alert or continues to sleep when the mother talks to him or the mother claps her hands or starts to undress him or stares blankly and appears not to see. If the child is not sleeping and is alert by your own observations, it is obvious that the child does not have this danger sign.

**Is the child able to sit or stand without support?** You should ask the mother if the infant child is able to sit and whether the older child is able to stand up. Ask the mother to place the child in a sitting or standing position and assess whether the child can sit or stand without support.

**LOOK**

*Explain to the CMDs:*

There are 4 symptoms that you need to look for in the child in the initial assessment to determine if the child has danger signs:

**Look for:**

- **Severe dehydration**: Check if the child has sunken eyes, coated tongue and is unable to drink
- **Severe anaemia or “lack of blood”**: Check if the child has pale lips or palms
- **Noisy or laboured breathing:** Listen if you can hear a grunting noise or stridor when the child breathes in or out, and whether the child seems to struggle to get air.

**If any of these danger signs are present, the child needs to be urgently referred to the nearest health facility.**

**If they are not present, continue to look for:**

**Cough or difficult breathing**

Ask the mother whether the child has cough or difficult breathing. If yes, ask “for how long?” Write how many days the child has had cough.

**Fast breathing**

If the child has cough or difficult breathing, use the timer to look for fast breathing as you were taught in section 2. Write down the number of breaths per minute. Remember the breathing cut-off rates are:

| 4 months up to 12 months - 50 or more breaths per minute  12 months to 5 years - 40 or more breaths per minute |
| --- |

**Chest indrawing:**

If the child has fast breathing according to the respiratory rate cutoffs, look for chest indrawing. Lay the child flat on his/her back and look if there is an inward movement of the chest whilst the child breathes in, whether the inward movement happens every time that the child breathes and whether this indrawing occurs even whilst the child is calm. If chest indrawing is present, the child needs to be urgently referred to the nearest health facility.

**If fast breathing is not present, the child is classified as having mild ARI**

**If fast breathing is present without chest indrawing the child is classified as having PNEUMONIA**

**If fast breathing and chest indrawing are present, refer to a health facility**

**FEEL**

**Fever**

If the caregiver reported a history of fever in the last 3 days as one of the child’s health problems you will need to measure the body temperature. Take temperature using the back of your hand. If the child has fever, or history of fever, record how many days since it started.

**If child has fever (reported by caregiver or hot on touch) and no fast breathing, the child is classified as having MALARIA ONLY.**

**If child has fever and fast breathing, the child is classified as having MALARIA and PNEUMONIA.**

## Classification of Illness for the 4 month to 5 year old children

As you will have noticed, the main ways to handle a child brought to you who is sick is a) to refer to a hospital or health centre, b) to give a medicine as treatment and c) to give advice on home care. It is important that the CMD appreciates how to classify a child into these groups.

*Explain to the CMDs:*

**A) Those to be referred to a hospital or health centre**:

- A child with any danger sign
- A sick child brought to you who is younger than 4 months or older than 5 years.
- A child who is brought to you who has other conditions such as cuts, diarrhoea, ear infection, fractures or poisoning
- A child whose illness you do not understand
- A child who has not improved despite treatment
- A child who is getting worse while on treatment
- A child who gets a serious bad medicine reaction to the medicine that you have given

**B) Those to be treated by the CMD:**

- A child with fast breathing and NO DANGER SIGN
- A child with fever and no fast breathing and NO DANGER SIGN

**C) All children treated by the CMD:**

- All children treated for fever and/or fast breathing

These classifications are provided in the fever diagnosis and management chart. First before reviewing the chart, we shall consider how the CMD should refer a child to the nearest hospital or health centre.

**PROCEDURE**

## 1) How to refer a child to a hospital or health centre

There are four main steps to take when you refer a child to the health facility. These are listed in the box 6 below

| **Box 6: Steps to take in referring a sick child to the nearest hospital or health centre**   Explain to the caretaker that the child is very sick and must be taken to the nearest hospital or health centre for treatment.   Assure the caretaker that the child will receive the best of care/treatment at the health center or hospital.   Give clear and specific instructions to caretakers regarding the care of the child on the way. That advice should include the following:   - 1. Clear secretions if nose is blocked.   2. Continue breastfeeding the young infant and increase fluids for the older child.    Write a referral note about the sick child. Give the referral note to the caretaker, who should carry it to the health worker at the health centre.   Ask the caretaker to come back to you with the referral card for feedback after it has been signed at the health facility |
| --- |

**Note:** Referring a child to a health unit does not mean that you have failed as a community health worker. Community members will appreciate your services better if you refer a child in time and save the child’s life than if you try to treat a very sick child and the child dies.

**PRACTICE**

**Role play**

Divide the CMDs into smaller groups of two or three. Have them practice completing the fever diagnosis chart using responses from their partners as a role play. The trainer should visit each group to evaluate understanding and make any necessary corrections. Each CMD should complete at least two charts or until they are comfortable with the chart.

**Case Scenarios**

Divide the CMDs into groups of three or four. Give the CMDs the following case histories to classify as either a) Refer to hospital or health centre, b) home care only, or c) Treatment by a CMD. The group should discuss the answers for each case. The group leader will present the results to the rest of the class.

| **Age** | **Symptoms** | **Correct classification** | **Coartem pack** | **Amoxi pack** |
| --- | --- | --- | --- | --- |
| 4 months | Cough, 40 breaths/min, fever | Home Therapy | Yellow | None |
| 1 year 3 months | Abnormally sleepy, Not able to drink, cough, 45 breaths/min, fever | Refer to a health facility | None | None |
| 2 years 6 months | Cough, 54 breaths/min, fever | Treatment by a CMD |  | Green |
| 3 years 4 months | Cough, Chest indrawing, 56 breaths/min, fever | Refer to Health facility | None | None |
| 4 years 3 days | Cough, runny nose, 36 breaths/min, fever | Treatment by a CMD | Blue | None |
| 6 months | Cough, 51 breaths/min, fever | Treatment by a CMD | Yellow | Pink |
| 4 years 7 months | Cough, 44 breaths/min, no fever | Treatment by CMD | None | Red |
| 3 years 4 months | Cough, 30 breaths/min, no fever | Home Therapy | None | None |

**Questions**

*Trainer should randomly ask CMDs the following questions to evaluate understanding of classification of illness for 4 month to 5 year olds:*

Q1. How would you classify patients aged 4 month to less than 5 years with fever?

Q2. Who should be treated by a CMD?

Q3. Who should be referred to a Health Facility?

Q4. How would you start to assess a child brought to you for treatment?

Q5. What advice do you give about care in the home

*Trainer should randomly ask CMDs the following questions to evaluate understanding of assessing a child for ARI:*

Q6. Give examples of specific question that you should ask the mother about the child?

Q7. What should you look for when assessing the child?

Q8. What should you feel for when assessing the child?

Q9. Why is it necessary to ask the child's age?

**2) How to give treatment will be considered in the next sections**

In order to provide a systematic way of assessing a child with fever and cough, making a diagnosis and deciding the treatment to give, the fever diagnosis and management chart has been designed as a tool for those CMDs in the intervention arm of the study.

**3) How to give advice on home care**

Home care involves the caregiver handling the sick child at home. The caregiver should give the treatment appropriately and look out for symptoms that indicate that the condition is getting worse. If this happens, they should return to the CMD or go to the nearest hospital or health centre. The advice to give is listed in the box 7 below

| **Box 7: Advice to give for the home care of a child**  The CMD should advise the caregiver to:   Give the full course of treatment as recommended   Clear secretions from the nose using cloth soaked in lukewarm water   Feed the child   Return to the CMD if child becomes sicker or is not able to drink or breastfeed |
| --- |

Treatment algorithm

| **ASK and LOOK** | **Signs present** | **Classification** | **ACTION** |
| --- | --- | --- | --- |
| ASK: What are the child’s problems? Tick each sign reported. If not reported, then ask to be sure. |  |  |  |
| Convulsions? | ⬜ Convulsions | Very severe illness | Refer to health facility |
| Altered mental state? | ⬜ altered mental state | Very severe illness | Refer to health facility |
| Not able to drink or breast feed? | ⬜ Not able to drink or breast feed | Very severe illness | Refer to health facility |
| Vomiting everything? | ⬜ Vomiting everything | Very severe illness | Refer to health facility |
| Severe dehydration | ⬜ Dehydration | Very severe illness | Refer to health facility |
| Abnormally sleepy or difficult to wake? | ⬜ Abnormally sleepy or difficult to wake | Very severe illness | Refer to health facility |
| Chest indrawing | ⬜ Chest indrawing | Very severe pneumonia | Refer to health facility |
| Extreme weakness | ⬜ Extreme weakness | Very severe illness | Refer to health facility |
| Severe anaemia | ⬜ Severe anaemia | Very severe illness | Refer to health facility |
| Noisy or laboured breathing | ⬜ Noisy breathing | Very severe illness | Refer to health facility |
| Cough or difficult breathing? If yes, for how long? ___ days  Count respiratory rate in 1 min:  ____ breaths/min | ⬜ Fast breathing | Pneumonia | Treat with Amoxicillin  Advise on home care |
|  | ⬜ No fast breathing | Cough or cold | Advise on home care |
| Fever or history of fever?  If yes, for how long? ___ days | ⬜ Fever | Malaria | Treat with Coartem  Advise on home care |

# Section 8 – How to fill the Registries and Referral Forms

Time: 4 hours

**OBJECTIVES**

By the end of the session the CMDs will be able to explain:

1. How to fill in patient record for classification and treatment of sick children
2. How to fill in referral forms for referred children
3. How to keep records

**MATERIALS**

CMD Job Aid, CMD register, referral form and adverse drug reaction form

**BACKGROUND**

As a CMD you will be required to keep good records. These pieces of information will be collected from you during supervision, when you go to collect medicines at the health facility and special visits by the study team. The records that you will need to keep are the following:

1. CMD register – this is form that you have been using but this version has been adjusted for this study.
2. Referral form – this is a new form that you will use when referring a child to the hospital or health centre
3. Bad medicine reaction form – this form is for recording the type of reactions that some children suffer from after taking Amoxi or Coartem.

**PROCEDURE for completing the CMD register**

You will be provided a copy of this register. Use this register for all children that you manage as part of your routine recording procedures. You will be explained how to complete this register taking note of the following:

 The register has columns for information on the name of the child, address, sex, age, main complaints, treatment, outcome, bad medicine reaction and referrals.

 This is a not a new register being introduced by the study.

 You already keep this register and you use information from it to complete the Monthly Aggregation form which you summit monthly to the Health Facility.

 To determine whether a child has received the pack “within 24 hours” or “after 24 hours” of fever onset, first ask the mother/caretaker how long the child has had fever.

 If the child receives (or mother/caretaker comes for) the pack before the same time the following day, take this to be “within 24 hours”

 If the child receives (or mother/caretaker comes for) the pack after the same time the following day, take this to be “after 24 hours”

 You can fill the outcome of treatment later when you make your visit to find out how the child is doing or when the caregiver reports back to you a few days later

 “Recovered” should be determined after 3 days when the treatment is completed.

 “Referred” should be filled at the time you refer the child.

 A child can have 2 different outcomes e.g. “referred” and “died” or “referred” and “recovered”.

**PRACTICE**

The trainer should mention some cases aloud for each CMD to practice filling into the register. Record this information on a flip chart with a marker. Give at least 5 examples and additional information that will allow the CMDs to complete the tally at the bottom. The trainer should go over each register to see how the CMD completed the columns. Identify any mistakes and discuss them with the class giving the necessary corrections.

**PROCEDURE for completing the referral form**

You will be provided with copies of the referral form. This form should be completed for every child that you refer to the nearest hospital or health centre. The form is divided into sections that will be explained to you. Remember to record following:

- Date and time of the referral
- Your name and address
- Name and age of child
- Main complaints and duration
- Sign the form.

The referral form will be a booklet with triplicate. The CMD should tear original and give it to the caregiver.

**CMD REFERRAL NOTE ­ Home management of malaria and pneumonia**

| **1. Referral Information by CMD** |  |
| --- | --- |
| CMD Code |  |
| Date of referral | dd /mm /yy |
| Name of Patient |  |
| Age of child in months |  |
| Sex of child 1=male, 2=female |  |
| Treatment given by CMD prior to referral:  1 = Coartem (colour) 2 = Amoxi (colour) |  |
| Date treatment was started | dd /mm /yy |
| Reason for referral:  1 = Severe Illness, 2 = Did not improve on treatment given,  3 = caregiver reported child getting worse, 4 = Other (specify) |  |
|  |  |
| **2. Referral Information (to be filled at Health facility)** |  |
| Date of arrival at health facility | dd /mm /yy |
| Time of arrival at health facility (24 hour clock) |  |
| Respiratory Rate on arrival |  |
| Microscopy results 1 = positive, 2 = negative, 3= Not available |  |
| Other laboratory tests done |  |
| Diagnosis made: |  |
| Patient admitted? 1 = Yes 2= No |  |
| If yes, date of admission |  |
| Date of discharge |  |
| **Treatment given and doses** |  |
|  |  |

Note {produce in triplicate and carbonated} 1- retained by CMD, 2-HF, 3-Caregiver

**PROCEDURE for completing the Adverse drug reaction reporting form**

# Section 9 – How to manage supplies

Time: 3 hours

**OBJECTIVES**

By the end of the session the CMDs will be able to explain:

1. How to keep the medicines
2. How to plan the drug stocks

**MATERIALS**

CMD Job Aid; stock card

**BACKGROUND**

## Keeping medicines

The quality of medicines will be badly affected if they are not properly kept. The medicines may no longer treat the sick children well and the community will loose trust in you. Therefore, items in stock should always be stored in a proper storage box. You will be provided with such a storage box. It is your duty to keep it and use it. The medicines should be kept in a cool part of the room, away from sunlight and from wet or damp surfaces.

**Keeping records of supplies**

Keeping records of the medicines and supplies that you have and use is a good practice. It can save you time and can protect you from false accusations. If you are accused of theft or misuse of supplies, you will be able to refer to your records. Your records will document the movement of supplies as you receive new quantities and as you use them. Managing supplies involves ordering, receiving, storing and issuing. It is important to keep good records of all of the medicines and supplies you receive so that you do not run out of medicines before you have had the chance to order more. Good record keeping helps you to know:

- What items are available in your stock
- The quantities of each item in your stock
- The quantities of each item that you use on a regular basis
- When and how much of an item you should reorder

**PROCEDURE**

To keep the medicines and supplies properly you should remember the following:

1. Keep the medicines in the storage box you have been provided at all times. Select a dry, cool and clean place in the house where the box should be kept. High temperatures and/or dampness cause the medicines to lose their strength.
2. Keep the medicines away from extremes of heat and cold.
3. Keep the medicines away from direct sunlight.
4. Keep the medicines separate from the other items in the house.
5. Do not allow children to play with the box and its contents.

For this study, the CMD will manage stocks of Amoxi and Coartem. You will have a stock card in which you will record information as shown below. You should complete this stock card once a week and before you order for more medicines or supplies. Take the cards with you when you go to the health centre for more medicines or whenever you go for supervision meetings.

## Stock card and dispensing record

To manage your stocks of medicines and supplies you should remember the following:

- Keep your stock card safely and always make sure that you have a sufficient number of them. If you run out of cards then use a note book making the columns above.
- Make weekly entries and before you order more medicines or supplies.
- Make a summary at the end of each month.
- Make a physical count at the end of the month to ensure the amount you have in stock equals the balance in your record.

**What the CMD should have as supplies**

Each CMD should have the following items:

- Enough stock of Amoxi for at least 1 month
- Enough stock of Coartem for at least 1 month
- A CMD register
- A pen
- An exercise book
- Job aids
- A box for storing medicines
- Stock cards
- A cup
- A spoon
- A jerrican
- A timer

**Stock card – Coartem**

**Name of CMD: ____________________________ Month:_______________**

**Village: ___________________________________ Year:______________**

**Maximum stock:__________**

**Minimum stock:__________**

Stock at end of Month: Yellow___________ Blue:______________

|  | **Stock-in** | | | **Stock-out** | |  |
| --- | --- | --- | --- | --- | --- | --- |
| Date | Package (yellow/blue) | Quantity | Supplier | Package (yellow/blue) | Quantity | Balance |
|  |  |  |  |  |  |  |
|  |  |  |  |  |  |  |
|  |  |  |  |  |  |  |
|  |  |  |  |  |  |  |
|  |  |  |  |  |  |  |
|  |  |  |  |  |  |  |
|  |  |  |  |  |  |  |
|  |  |  |  |  |  |  |
|  |  |  |  |  |  |  |
|  |  |  |  |  |  |  |
|  |  |  |  |  |  |  |
|  |  |  |  |  |  |  |
|  |  |  |  |  |  |  |
|  |  |  |  |  |  |  |
|  |  |  |  |  |  |  |
|  |  |  |  |  |  |  |
|  |  |  |  |  |  |  |
|  |  |  |  |  |  |  |
|  |  |  |  |  |  |  |
|  |  |  |  |  |  |  |
|  |  |  |  |  |  |  |
|  |  |  |  |  |  |  |
|  |  |  |  |  |  |  |
|  |  |  |  |  |  |  |
|  |  |  |  |  |  |  |
|  |  |  |  |  |  |  |
|  |  |  |  |  |  |  |
|  |  |  |  |  |  |  |
|  |  |  |  |  |  |  |
|  |  |  |  |  |  |  |
|  |  |  |  |  |  |  |
|  |  |  |  |  |  |  |
|  |  |  |  |  |  |  |
|  |  |  |  |  |  |  |
|  |  |  |  |  |  |  |
|  |  |  |  |  |  |  |
|  |  |  |  |  |  |  |
|  |  |  |  |  |  |  |

**Stock card – Amoxi**

**Name of CMD: ____________________________ Month:_______________**

**Village: ___________________________________ Year:______________**

**Maximum stock:__________**

**Minimum stock:__________**

Stock at end of Month: Pink___________ Green:________ Red:__________

|  | **Stock-in** | | | **Stock-out** | |  |
| --- | --- | --- | --- | --- | --- | --- |
| Date | Package (pink/green/red) | Qty | Supplier | Package (pink/green/red) | Qty | Balance |
|  |  |  |  |  |  |  |
|  |  |  |  |  |  |  |
|  |  |  |  |  |  |  |
|  |  |  |  |  |  |  |
|  |  |  |  |  |  |  |
|  |  |  |  |  |  |  |
|  |  |  |  |  |  |  |
|  |  |  |  |  |  |  |
|  |  |  |  |  |  |  |
|  |  |  |  |  |  |  |
|  |  |  |  |  |  |  |
|  |  |  |  |  |  |  |
|  |  |  |  |  |  |  |
|  |  |  |  |  |  |  |
|  |  |  |  |  |  |  |
|  |  |  |  |  |  |  |
|  |  |  |  |  |  |  |
|  |  |  |  |  |  |  |
|  |  |  |  |  |  |  |
|  |  |  |  |  |  |  |
|  |  |  |  |  |  |  |
|  |  |  |  |  |  |  |
|  |  |  |  |  |  |  |
|  |  |  |  |  |  |  |
|  |  |  |  |  |  |  |
|  |  |  |  |  |  |  |
|  |  |  |  |  |  |  |
|  |  |  |  |  |  |  |
|  |  |  |  |  |  |  |
|  |  |  |  |  |  |  |
|  |  |  |  |  |  |  |
|  |  |  |  |  |  |  |
|  |  |  |  |  |  |  |
|  |  |  |  |  |  |  |
|  |  |  |  |  |  |  |
|  |  |  |  |  |  |  |
|  |  |  |  |  |  |  |

##### TREATMENT RECORDING FORM – COMMUNITY MEDICINE DISTRIBUTOR’S REGISTER

Month __________________Year _____________________ Name of Community Medicine Distributor_________________________ ______

Village (LCI) ____________ Parish (LCII) ____________ Sub-County (LCIII) ____________ HSD ____________ District_____________

| Date (dd/mm/yyyy) | Names of child | Sex (F/M) | Age  (yrs/mo) | Name of mother/caretaker & relationship | Danger signs | Respiratory  Rate  (breaths/  min) | COARTEM pack given  (Yellow, Blue or None) | Amoxicillin pack given (Pink, green, red) | Interval from fever onset  (Tick √) | | Outcome (Tick√) | | | Bad Medicine Reaction | Slept under  mosquito net  last night |
| --- | --- | --- | --- | --- | --- | --- | --- | --- | --- | --- | --- | --- | --- | --- | --- |
|  |  |  |  |  |  |  |  |  | Within 24 hrs | After 24 hrs | Referred | Recovered | Died | (Yes/No) | (Yes/No) |
|  |  |  |  |  |  |  |  |  |  |  |  |  |  |  |  |
|  |  |  |  |  |  |  |  |  |  |  |  |  |  |  |  |
|  |  |  |  |  |  |  |  |  |  |  |  |  |  |  |  |
|  |  |  |  |  |  |  |  |  |  |  |  |  |  |  |  |
|  |  |  |  |  |  |  |  |  |  |  |  |  |  |  |  |
|  |  |  |  |  |  |  |  |  |  |  |  |  |  |  |  |
|  |  |  |  |  |  |  |  |  |  |  |  |  |  |  |  |
|  |  |  |  |  |  |  |  |  |  |  |  |  |  |  |  |
|  |  |  |  |  |  |  |  |  |  |  |  |  |  |  |  |
|  |  |  |  |  |  |  |  |  |  |  |  |  |  |  |  |
|  |  |  |  |  |  |  |  |  |  |  |  |  |  |  |  |
|  |  |  |  |  |  |  |  |  |  |  |  |  |  |  |  |
|  |  |  |  |  |  |  |  |  |  |  |  |  |  |  |  |
|  |  |  |  |  |  |  |  |  |  |  |  |  |  |  |  |
|  |  |  |  |  |  |  |  |  |  |  |  |  |  |  |  |
|  |  |  |  |  |  |  |  |  |  |  |  |  |  |  |  |
|  |  |  |  |  |  |  |  |  |  |  |  |  |  |  |  |
|  |  |  |  |  |  |  |  |  |  |  |  |  |  |  |  |

| Tally/cross | | | |
| --- | --- | --- | --- |
| Births during the month | M | OOOOO OOOOO OOOOO OOOOO OOOOO OOOOO OOOOO OOOOO OOOOO | |
|  | F | OOOOO OOOOO OOOOO OOOOO OOOOO OOOOO OOOOO OOOOO OOOOO | |
| Children less than 5 years not treated by CMDs who died | M | OOOOO OOOOO OOOOO OOOOO OOOOO OOOOO OOOOO OOOOO OOOOO | |
|  | F | OOOOO OOOOO OOOOO OOOOO OOOOO OOOOO OOOOO OOOOO OOOOO | |
| Children from 5 to 7 years of age not treated by CMDs who died | M | OOOOO OOOOO OOOOO OOOOO OOOOO OOOOO OOOOO OOOOO OOOOO | |
|  | F | OOOOO OOOOO OOOOO OOOOO OOOOO OOOOO OOOOO OOOOO OOOOO | |
| Were you supervised by health facility staff this month? | Yes |  |  |
|  | No |  |  |
